# Supplementary material for: Treatment- and population-specific genetic risk factors for anti-drug antibodies against interferon-beta: a GWAS
Source: BMC Med. 2020 Nov 4;18:298. doi: 10.1186/s12916-020-01769-6 (PMC7641861; doi:10.1186/s12916-020-01769-6)
Supplement: Supplementary file 1 — Additional file 1. Previous measurements and design of new ADA measurements. Previous ADA measurements in the Swedish KI and German TUM cohorts per treatment preparation and distribution of samples for the new ADA measurements. For part of the TUM patients, only previous bADA measurements were available. [file 12916_2020_1769_MOESM1_ESM.pdf]

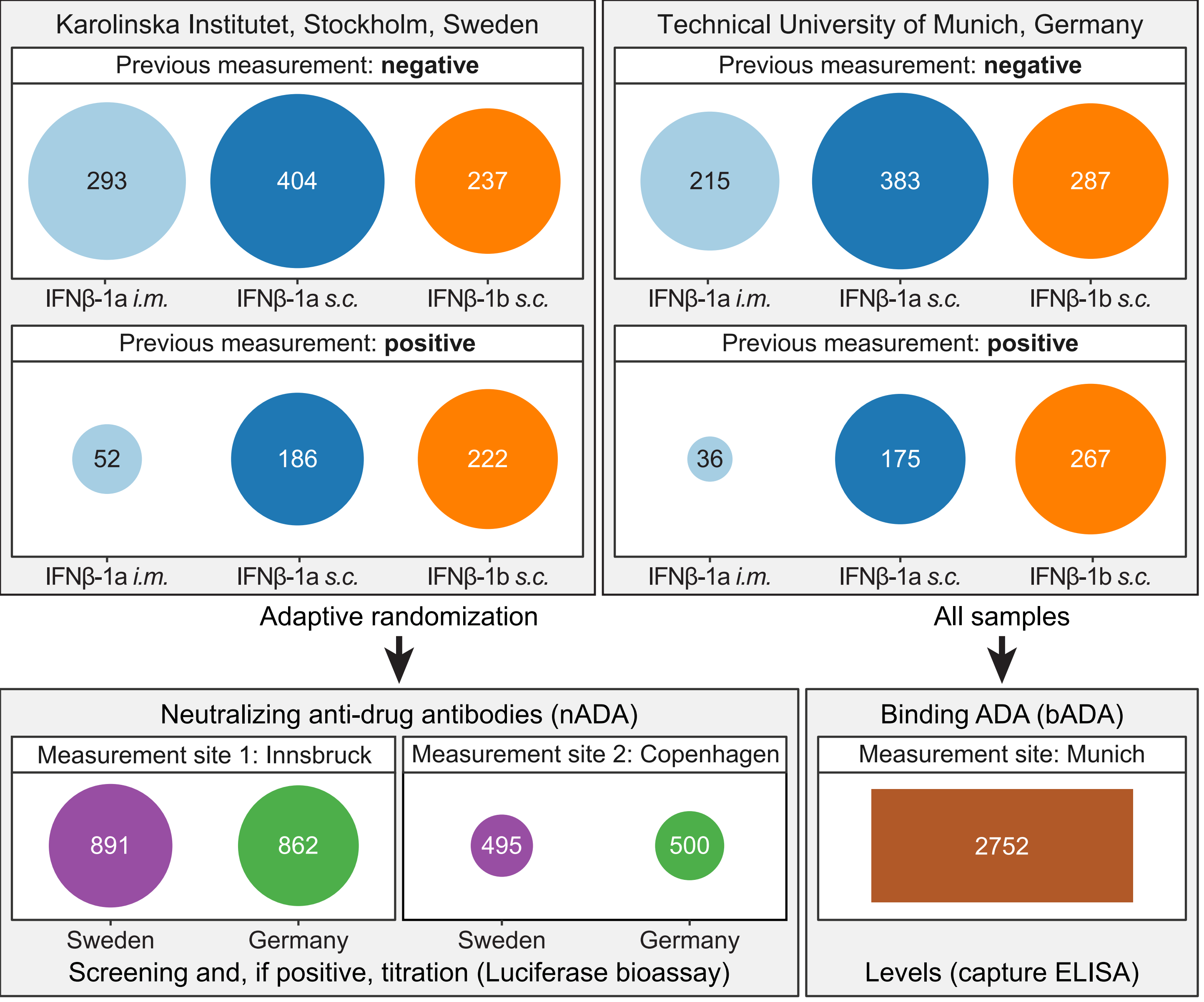
Karolinska Institutet, Stockholm, Sweden
Previous measurement: negative
293
404
237
IFNβ-1a i.m.
IFNβ-1a s.c.
IFNβ-1b s.c.
Previous measurement: positive
52
186
222
IFNβ-1a i.m.
IFNβ-1a s.c.
IFNβ-1b s.c.
Technical University of Munich, Germany
Previous measurement: negative
215
383
287
IFNβ-1a i.m.
IFNβ-1a s.c.
IFNβ-1b s.c.
Previous measurement: positive
36
175
267
IFNβ-1a i.m.
IFNβ-1a s.c.
IFNβ-1b s.c.
Adaptive randomization
All samples
Neutralizing anti-drug antibodies (nADA)
Measurement site 1: Innsbruck
891
862
Sweden
Germany
Measurement site 2: Copenhagen
495
500
Sweden
Germany
Screening and, if positive, titration (Luciferase bioassay)
Binding ADA (bADA)
Measurement site: Munich
2752
Levels (capture ELISA)
