# Supplementary material for: Treatment- and population-specific genetic risk factors for anti-drug antibodies against interferon-beta: a GWAS
Source: BMC Med. 2020 Nov 4;18:298. doi: 10.1186/s12916-020-01769-6 (PMC7641861; doi:10.1186/s12916-020-01769-6)
Supplement: Supplementary file 2 — Additional file 2. Additional details supporting the Methods section [file 12916_2020_1769_MOESM2_ESM.pdf]

Additional Methods

Quantification of bADA

Binding ADA levels were assessed using capture ELISA. Levels were calculated from optical densities (OD) using a standard curve. Dilutions for the standard curve were 1:100, 1:200, 1:400, 1:800, 1:1600, 1:3200, 1:6400, and 1:12800. For each microtiter plate *n*, the coefficients *a*, *b*, *c*, and *d* were estimated in a linear model using the following formula:

$$log\left(\frac{1}{dilution}\right) \sim a + b \times \log(OD_{standard\ n}) + c \times \log(OD_{standard\ n})^2 + d \times \log(OD_{standard\ n})^3$$

These plate-specific coefficients were then used for the calculation of levels from ODs:

$$level = exp(a + b \times \log(OD_n) + c \times \log(OD_n)^2 + d \times \log(OD_n)^3) \times 100{,}000$$

For one measurement with a negative OD, the level was set to 0.
Levels were normalized for analyses in linear regression models by rank-based inverse normal transformation, separately for the discovery and replication datasets.
To assess the reliability of the ELISA, 706 samples were assayed a second time; the Spearman correlation coefficient between both measurements was ρ=0.90. Only the original (first) measurements were analyzed in the present study.

Titration of nADA

In Copenhagen, nADA titers were determined to a dilution of 1:20,480. In Innsbruck, nADA titers were assessed to a maximum dilution of 1:5,120. To avoid batch effects caused by this methodological difference, n=89 samples with titers >5,120 in the Copenhagen dataset were capped to 5,120. The final dataset contained 116 samples (11.7 %) with a titer of 1:5,120 from Copenhagen and 108 samples (6.1 %) with a titer of 1:5,120 from Innsbruck. The measurement site was used as a covariate in all analyses of nADA titers or presence.

Estimation of the nADA status from bADA levels

The nADA cutoff of the bADA level was estimated in the discovery data, with the aim of maximizing sensitivity and specificity. The optimal cutoff was determined using the *MaxSpSe* method of the *R* package *OptimalCutpoints*. To avoid overfitting the cutoff, nested cross-validation was applied with three outer and four inner folds. In each outer cross-validation instance, the cutoff producing the maximum sensitivity across three inner cross-validation folds was tested on the remaining fold. Nested cross-validation was repeated 100 times and the mean cutoff of the 100 repetitions was used as the final cutoff and tested on the replication dataset.

Concordance between previous and new nADA measurements

For 90.2% of our sample, a previous assessment of the presence of nADA was available. Note that the methods used for measuring nADA previously differed from the methods used in the present study. To assess the concordance between the old and new measurements of the presence of nADA, we calculated Cohen's Kappa using the function *kappa2* of the *R* package *irr*. The results were as follows:

Entire dataset: κ = 0.742
KI Sweden: κ = 0.812
TUM Germany: κ = 0.637

Genetic quality control (QC) and imputation

QC of genotype data was conducted separately per cohort in PLINK v1.90b3.36 (or higher) and *R* v3.3.3. For the TUM dataset, QC was carried out first on each of both datasets separately (Illumina OmniExpress and Human660-Quad), followed by a second round of QC on the combined dataset.

Sequence of genotype QC

1.1. Removal of SNPs with call rates <98% or a minor allele frequency (MAF) <1%
1.2. Removal of individuals with genotyping rates <98%
1.3. Removal of sex mismatches
1.4. Removal of genetic duplicates
1.5. Removal of cryptic relatives with PI-HAT≥12.5
1.6. Removal of genetic outliers with a distance from the mean of >4 SD in the first eight MDS (ancestry) components
1.7. Removal of individuals with a deviation of the autosomal or X-chromosomal heterozygosity from the mean >4 SD
1.8. Removal of non-autosomal variants
1.9. Removal of SNPs with call rates <98%, a MAF <1%, or Hardy-Weinberg Equilibrium (HWE) test *p*-values $<1\times10^{-6}$
1.10. Removal of A/T and G/C SNPs
1.11. Update of variant IDs and positions to the IDs and positions in the 1000 Genomes Phase 3 reference panel
1.12. Alignment of alleles to the reference panel
1.13. Removal of duplicated variants and variants not present in the reference panel

Datasets

1. TUM Illumina OmniExpress
   - Before QC: 1,071 individuals and 729,801 variants
   - After QC: 992 individuals and 607,230 variants
2. TUM Illumina Human660-Quad
   - Before QC: 708 individuals and 593,392 variants
   - After QC: 693 individuals and 530,199 variants
3. TUM combined
   - Before QC: 1,685 individuals and 293,486 variants
   - After QC: 1,675 individuals and 293,418 variants
   - After imputation: 8,550,834 variants
4. KI Illumina OmniExpress
   - Before QC: 2,225 individuals and 716,503 variants
   - After QC: 1,896 individuals and 609,187 variants
   - After imputation: 9,096,778 variants

Imputation of genotype data

Genotypes were aligned to the 1000 Genomes Phase 3 reference panel using SHAPEIT v2 (r837) and PLINK. Pre-phasing (haplotype estimation) was conducted for each chromosome separately using SHAPEIT. Imputation was performed using IMPUTE2 v2.3.2 in 5 Mbp chunks with 500 kbp buffers, filtering out variants that are monomorphic in the EUR samples. Chunks with <51 genotyped variants or concordance rates <92 % were fused with neighboring chunks and re-imputed. Variants with a MAF <1% or an INFO metric <0.8 were removed after imputation.

Imputation of *HLA* alleles

HLA allele imputation was performed using SNP2HLA v1.0.3 / Beagle v3.04 and the Type 1 Diabetes Genetics Consortium imputation panel, as previously described. After filtering for an allele frequency of ≥1% and a Beagle imputation $r^2 \geq 0.3$, If a 4-digit and a corresponding 2-digit allele showed an LD $r^2 > 0.99$, the two-digit allele was excluded from analyses. In total, 122 *HLA* alleles were analyzed.

In addition, the following nine extended haplotypes (Horton *et al.*, 2008) and their sub-haplotypes were determined from Beagle phasing results and analyzed:

A3-B7-DR15-DQ6 (A3-DQ6):
*HLA A*0301 : C*0702 : B*0702 : DRB1*1501 : DQA1*0102 : DQB1*0602*
C7-DR15-DQ6 (C7-DQ6):
*C*0702 : B*0702 : DRB1*1501 : DQA1*0102 : DQB1*0602*

B7-DR15-DQ6 (B7-DQ6):
*B*0702 : DRB1*1501 : DQA1*0102 : DQB1*0602*
DR15-DQ6 (DR15-DQ6):
*DRB1*1501 : DQA1*0102 : DQB1*0602*

A1-B8-DR3-DQ2: (A1-DQ2)
*HLA A*0101 : C*0701 : B*0801 : DRB1*0301 : DQA1*0501 : DQB1*0201*
C7-DR3-DQ2: (C7-DQ2)
*C*0701 : B*0801 : DRB1*0301 : DQA1*0501 : DQB1*0201*
B8-DR3-DQ2: (B8-DQ2)
*B*0801 : DRB1*0301 : DQA1*0501 : DQB1*0201*
DR3-DQ2: (DR3-DQ2)
*DRB1*0301 : DQA1*0501 : DQB1*0201*

DR4-DQ3: (DR4-DQ3)
*DRB1*0401 : DQA1*0301 : DQB1*0302*

Calculation of MDS ancestry components

For the calculation of multidimensional scaling (MDS) ancestry components (population stratification), pre-imputation genotype data was used, after the QC steps explained above had been applied. Additional variant filtering steps were: removal of variants with a MAF <0.05 or HWE *p*-value $<10^{-3}$; removal of variants mapping to the extended MHC region (chromosome 6, 25-35 Mbp) or to a typical inversion site on chromosome 8 (7-13 Mbp); LD pruning (command *--indep-pairwise 200 100 0.2*). Next, the pairwise identity-by-state (IBS) matrix of all individuals was calculated using the command *--genome* on the filtered genotype data. The MDS analysis was performed on the IBS matrix using the eigendecomposition-based algorithm in PLINK. MDS components calculated for each cohort separately were used as covariates (the ancestry components) in analyses using genetic data.

Estimation of the SNP heritability

The GCTA genome-based restricted maximum likelihood (GREML) method was used to estimate the SNP heritability h^2_g. To this end, the genotype data after QC (but before imputation) from both cohorts (Sweden and Germany) was merged in PLINK. In the merged dataset, only variants present in the HapMap r28 CEU release, with a call rate ≥0.95, a HWE *p*-value $\geq 10^{-6}$, and a MAF ≥0.01 were retained. Cohort, sex, age, treatment type, treatment duration, eight MDS ancestry components, and, for nADA titers and status, titration site (Innsbruck / Copenhagen), were used as covariates. GREML was conducted using the option *--grm-adj 0*, assuming that causal variants have a similar distribution of allele frequencies as the genotyped variants.

GWAS

SNPs were prioritized for replication based on the following criteria:

1. Genome-wide significance ($p<5\times10^{-8}$) in the discovery-stage GWAS
2. SNP with the lowest *p*-value within 100,000 bp
3. SNP with LD $r^2<0.2$ with other, more strongly associated SNPs in each cohort

Although the GWAS were conducted in PLINK, followed by meta-analysis in METAL, all results of prioritized variants presented in the main and supplemental tables have been calculated in *R*, to avoid minor errors caused by automatic rounding to four digits in PLINK. This rounding leads to a lack in precision especially for logistic regression, where the four-digit rounded ORs are converted to log(OR) effect sizes before the two-step (first by country, then by treatment) meta-analyses.

Generation and analysis of polygenic risk scores (PRS)

Discovery-phase summary statistics (training data) were clumped in PLINK, based on best-guess genotype data (hard-call threshold 0.3) using the following parameters:
--clump-kb 500 --clump-r2 0.1 --clump-p1 1 --clump-p2 1
PRS were then calculated in R v.3.3 based on imputed (dosage) data. Test statistics and alleles in the GWAS training data were flipped so that effect sizes pointed in a positive direction. Thus, the PRS represent weighted, cumulative, additive risk. For each disorder, we calculated eight cumulative PRS (sum of risk load) with different *p*-value thresholds: $<5\times10^{-8}$, $<1\times10^{-7}$, $<1\times10^{-6}$, $<1\times10^{-5}$, $<1\times10^{-4}$, <0.001, <0.01, <0.05. PRS were scaled to represent relative risk load (minimum possible cumulative risk load = 0, maximum = 1). For each analysis, we selected the threshold showing the strongest association.
