# Supplementary material for: Treatment- and population-specific genetic risk factors for anti-drug antibodies against interferon-beta: a GWAS
Source: BMC Med. 2020 Nov 4;18:298. doi: 10.1186/s12916-020-01769-6 (PMC7641861; doi:10.1186/s12916-020-01769-6)
Supplement: Supplementary file 3 — Additional file 3. New ADA measurements and design of the datasets for analyses. New ADA measurements in the Swedish KI and German TUM cohorts per treatment preparation and assignments of samples into the discovery and replication datasets. In the discovery and replication datasets, the first number indicates nADA and the second number bADA measurements. The distinction into negative and positive patients was made using nADA measurements. [file 12916_2020_1769_MOESM3_ESM.pdf]

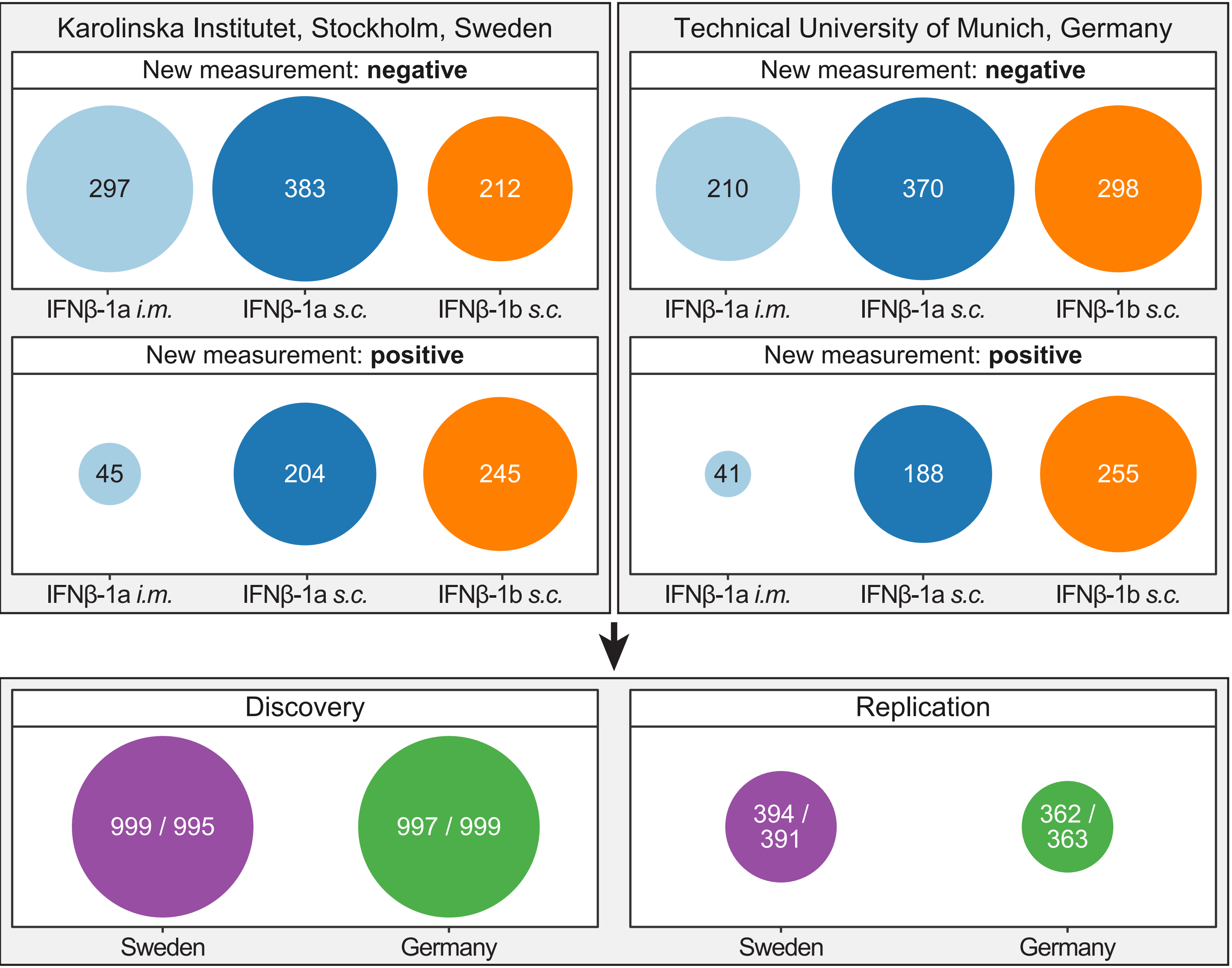
Karolinska Institutet, Stockholm, Sweden
New measurement: negative
297
383
212
IFNβ-1a i.m.
IFNβ-1a s.c.
IFNβ-1b s.c.
New measurement: positive
45
204
245
IFNβ-1a i.m.
IFNβ-1a s.c.
IFNβ-1b s.c.
Technical University of Munich, Germany
New measurement: negative
210
370
298
IFNβ-1a i.m.
IFNβ-1a s.c.
IFNβ-1b s.c.
New measurement: positive
41
188
255
IFNβ-1a i.m.
IFNβ-1a s.c.
IFNβ-1b s.c.
Discovery
999 / 995
997 / 999
Sweden
Germany
Replication
394 / 391
362 / 363
Sweden
Germany
