# Supplementary material for: Treatment- and population-specific genetic risk factors for anti-drug antibodies against interferon-beta: a GWAS
Source: BMC Med. 2020 Nov 4;18:298. doi: 10.1186/s12916-020-01769-6 (PMC7641861; doi:10.1186/s12916-020-01769-6)

Population stratification

We calculated multidimensional scaling (MDS) ancestry components on quality-controlled genotype data by carrying out the following steps:

1. Removal of variants meeting the following criteria:
   - Minor allele frequency (MAF) <0.05
   - Hardy-Weinberg equilibrium test (HWE) *p*-value $<10^{-3}$
   - Variants mapping to the extended MHC region (chromosome 6, 25-35 Mbp)
   - Variants mapping to a typical inversion site on chromosome 8 (7-13 Mbp)
2. Linkage disequilibrium (LD) pruning in PLINK by using the command `--indep-pairwise 200 100 0.2`
3. Calculation of the pairwise identity-by-state (IBS) matrix of all individuals using the command `--genome` on the filtered and pruned genotype data.
4. The MDS analysis was performed on the IBS matrix using the eigendecomposition-based algorithm in PLINK using the command: `--cluster --mds-plot 10 eigendecomp`

There were no significant differences in the MDS ancestry components between the discovery and replication datasets of the two cohorts (*p*-values calculated using Student's t-tests):

Component	*p* KI Sweden	*p* TUM Germany
**C1**	0.578543	0.83383
**C2**	0.115316	0.154049
**C3**	0.148339	0.335012
**C4**	0.434946	0.688109
**C5**	0.354306	0.18717
**C6**	0.794402	0.811636
**C7**	0.116679	0.478874
**C8**	0.0803	0.05891

A: Population substructure on the merged genotype data of both cohorts (magenta: KI Sweden, green: TUM Germany), visualized by MDS ancestry components, calculated as described above. The axes have been scaled to standard deviations. Both cohorts are clearly separated along the first component, indicating population differences between both samples.

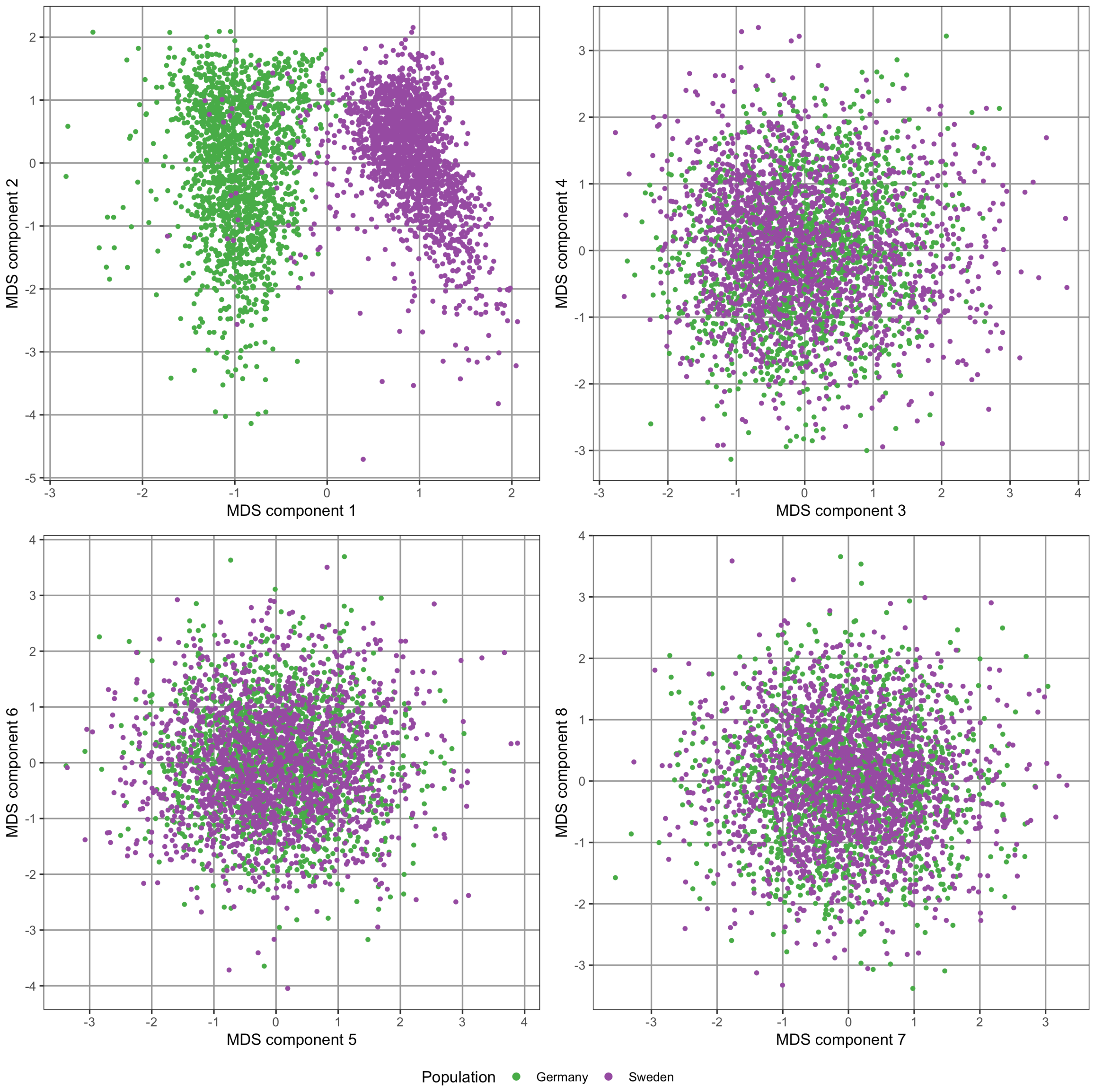

B: Population substructure of the KI Sweden sample, visualized by MDS ancestry components. The axes have been scaled to standard deviations. No perceptible subpopulations or significant genetic outliers can be observed.

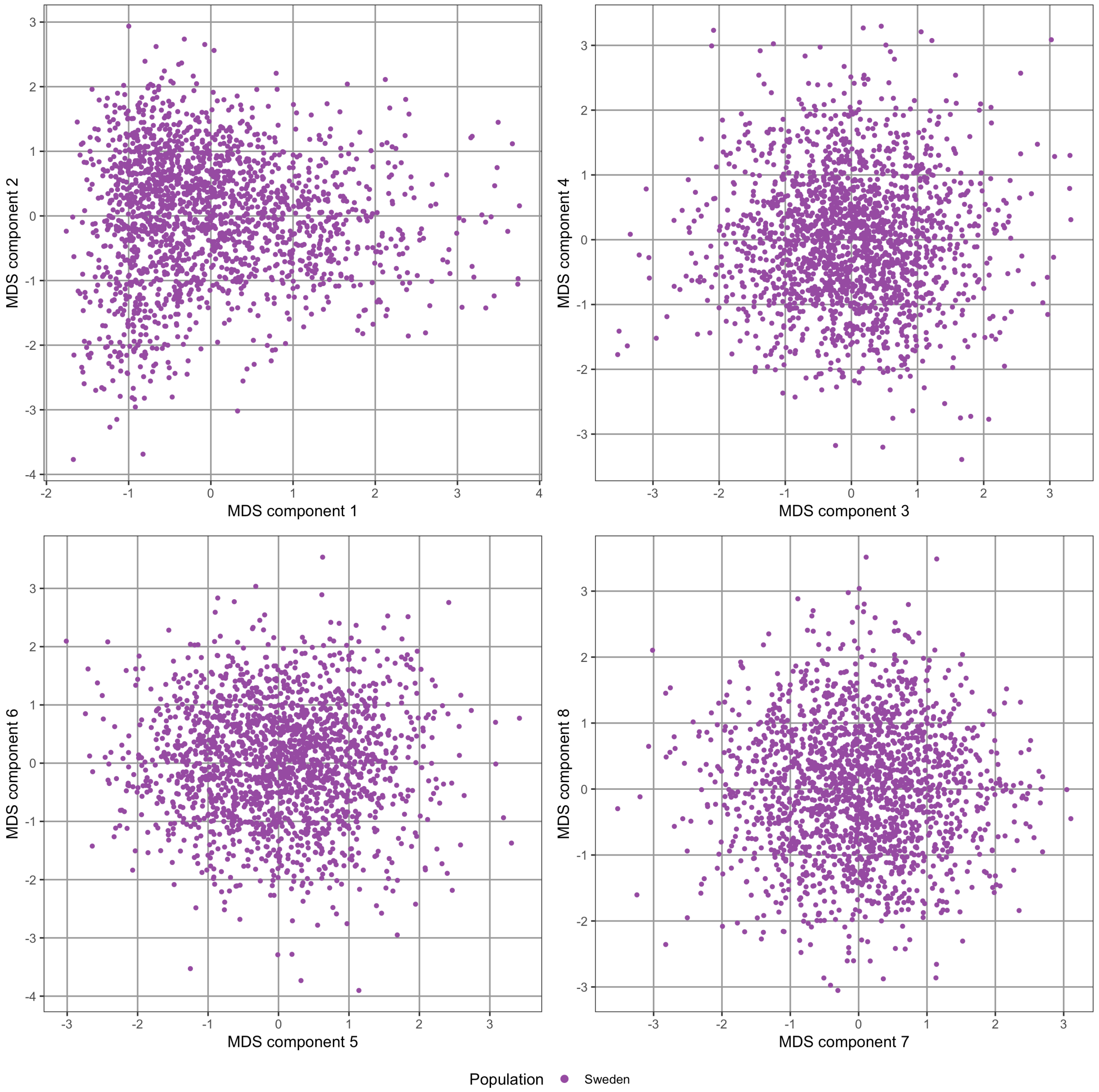

C: Population substructure of the TUM Germany sample, visualized by MDS ancestry components. The axes have been scaled to standard deviations. No perceptible subpopulations or significant genetic outliers can be observed.

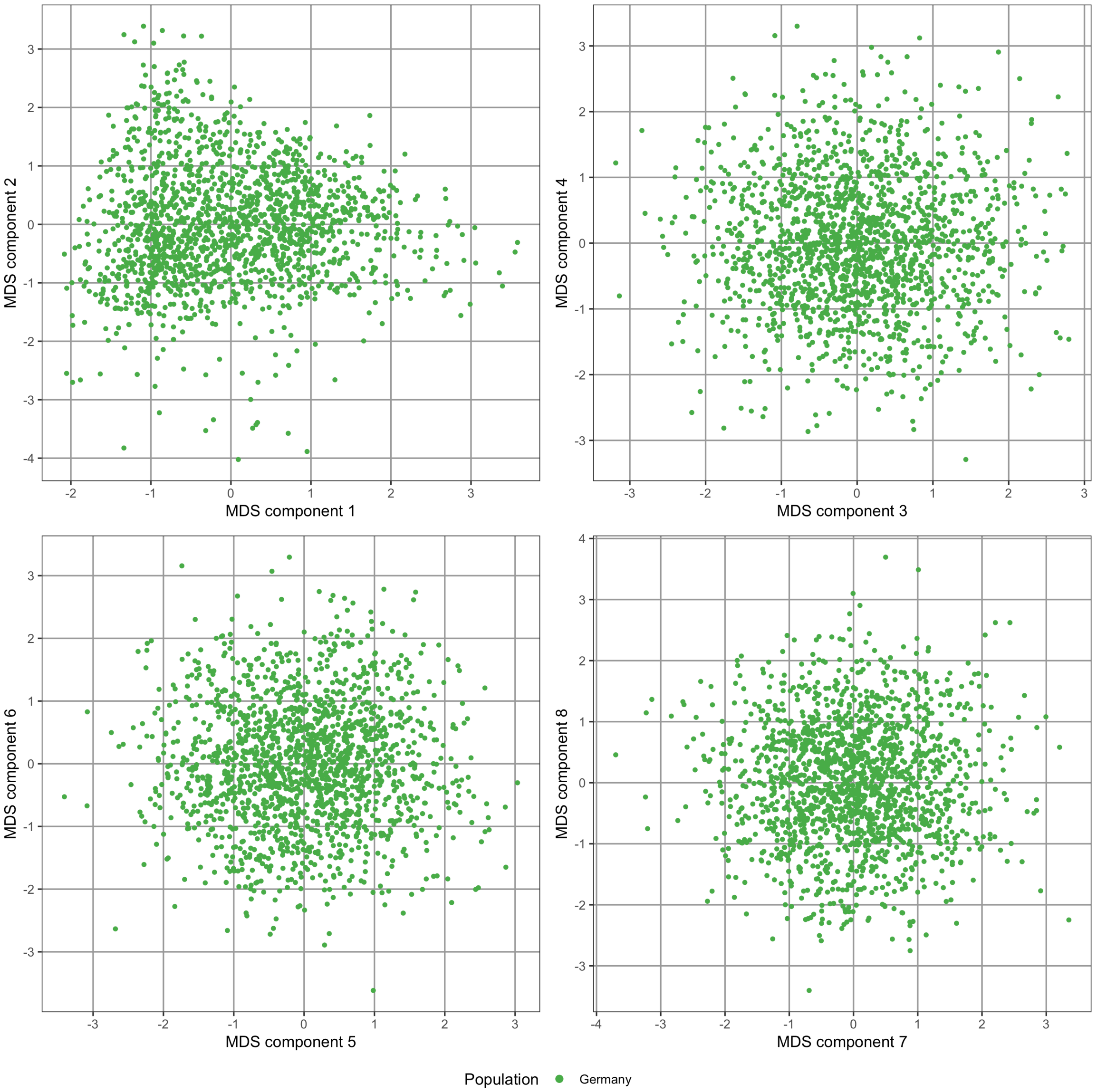

D: The distributions of individuals genotyped on the two microarray types used in the TUM Germany cohort overlap. The plot shows MDS ancestry components with axes scaled to standard deviations.

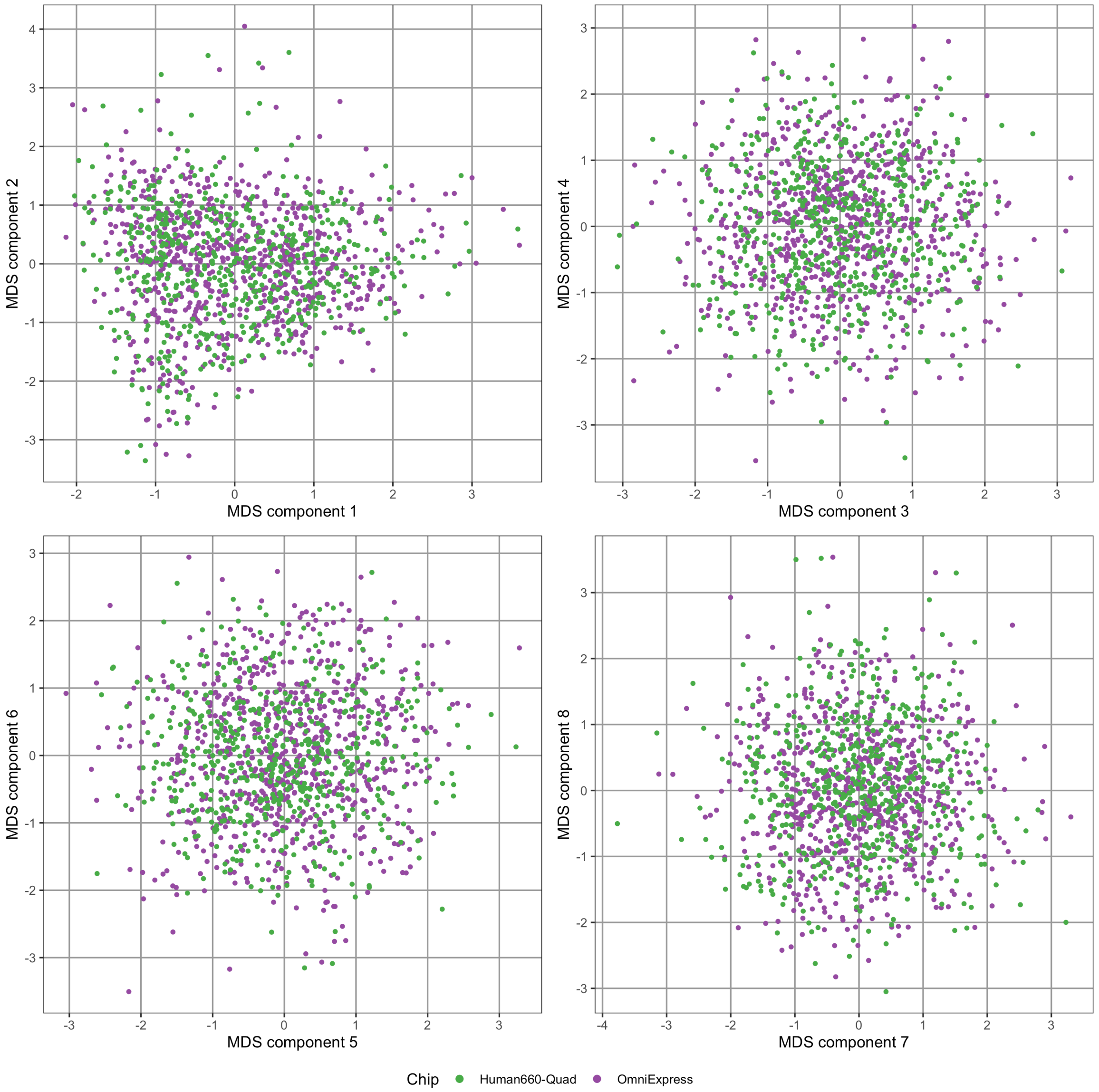

Supplement: Supplementary file 4 — Additional file 4. Visualization and analysis of population stratification. For detailed figure legends, see the file. [file 12916_2020_1769_MOESM4_ESM.pdf]
