# Supplementary figures and images for: Treatment- and population-specific genetic risk factors for anti-drug antibodies against interferon-beta: a GWAS
Source: BMC Med. 2020 Nov 4;18:298. doi: 10.1186/s12916-020-01769-6 (PMC7641861; doi:10.1186/s12916-020-01769-6)

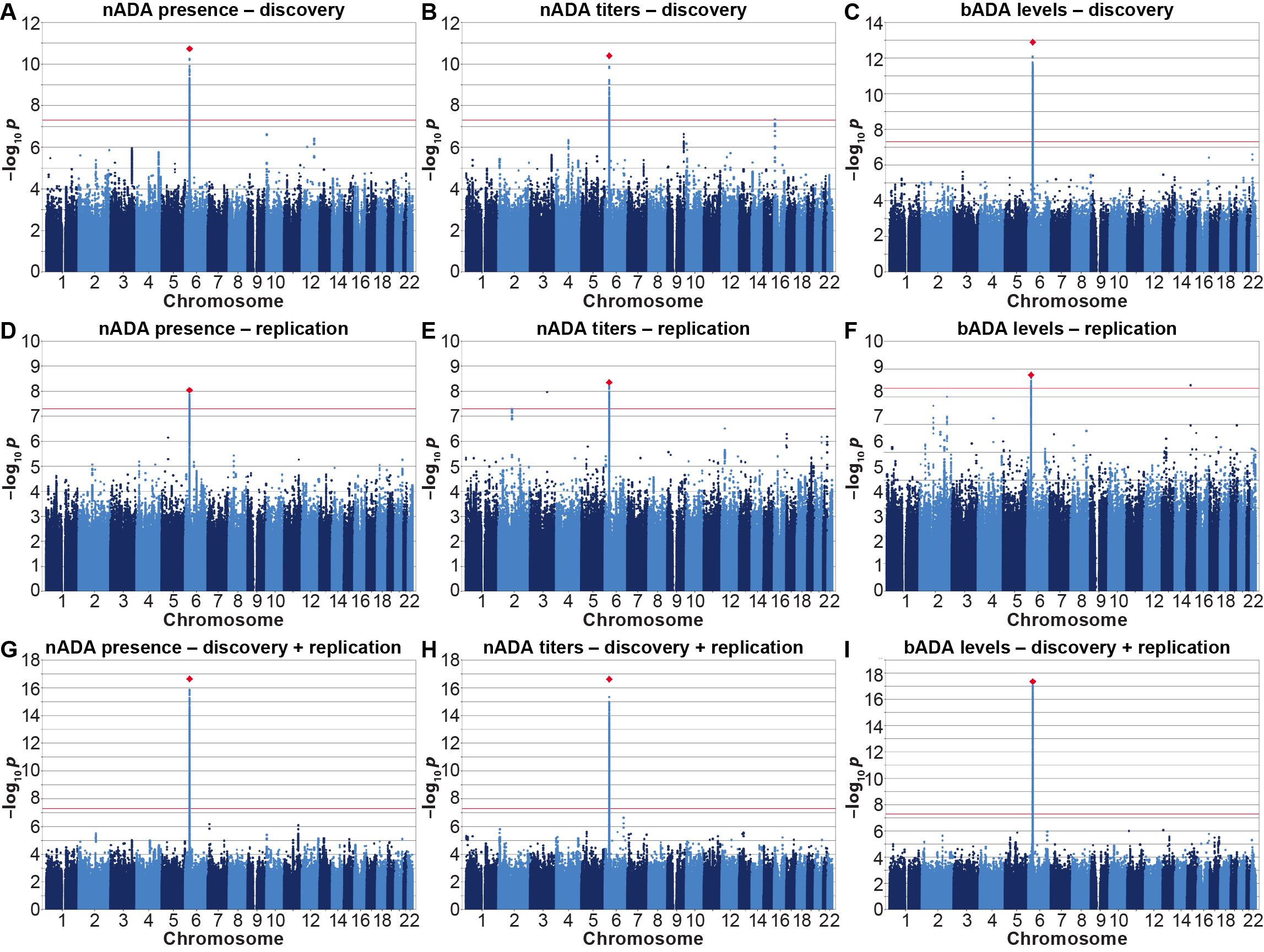

Supplement: Supplementary file 5 — Additional file 5. Manhattan plots of the GWAS across IFNβ preparations. Manhattan plots of the (A-C) discovery-stage, (D-F) replication-stage, and (G-I) pooled discovery + replication GWAS. The red line between -log10p = 7 and -log10p = 8 indicates genome-wide significance; the top genome-wide significant variant is labeled with a red diamond. [file 12916_2020_1769_MOESM5_ESM.png]

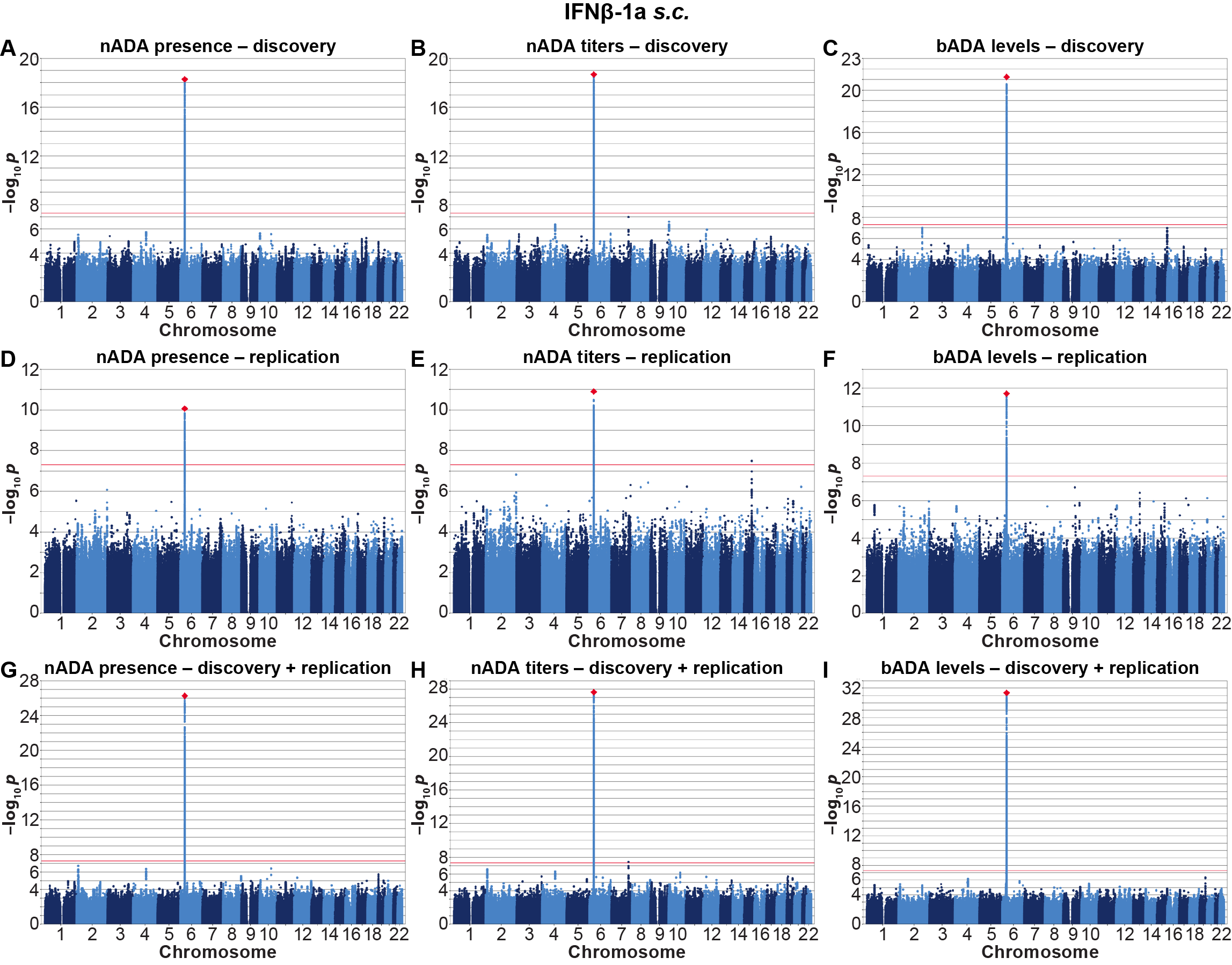

Supplement: Supplementary file 6 — Additional file 6. Manhattan plots of the GWAS on patients treated with IFNβ-1a s.c. Manhattan plots of the (A-C) discovery-stage, (D-F) replication-stage, and (G-I) pooled discovery + replication GWAS. The red line between -log10p = 7 and -log10p = 8 indicates genome-wide significance; the top genome-wide significant variant is labeled with a red diamond. [file 12916_2020_1769_MOESM6_ESM.png]

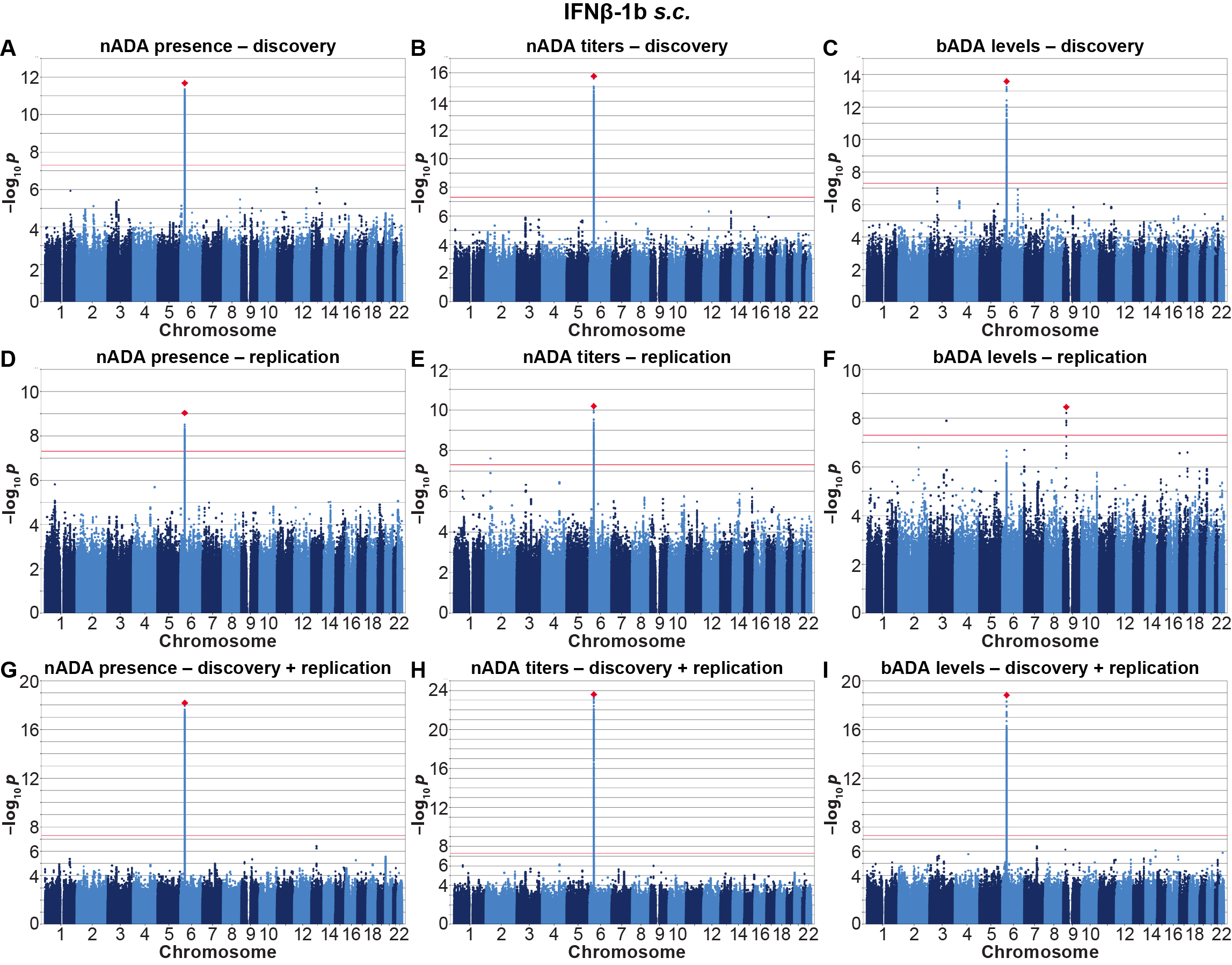

Supplement: Supplementary file 7 — Additional file 7. Manhattan plots of the GWAS on patients treated with IFNβ-1b s.c. Manhattan plots of the (A-C) discovery-stage, (D-F) replication-stage, and (G-I) pooled discovery + replication GWAS. The red line between -log10p = 7 and -log10p = 8 indicates genome-wide significance; the top genome-wide significant variant is labeled with a red diamond. [file 12916_2020_1769_MOESM7_ESM.png]

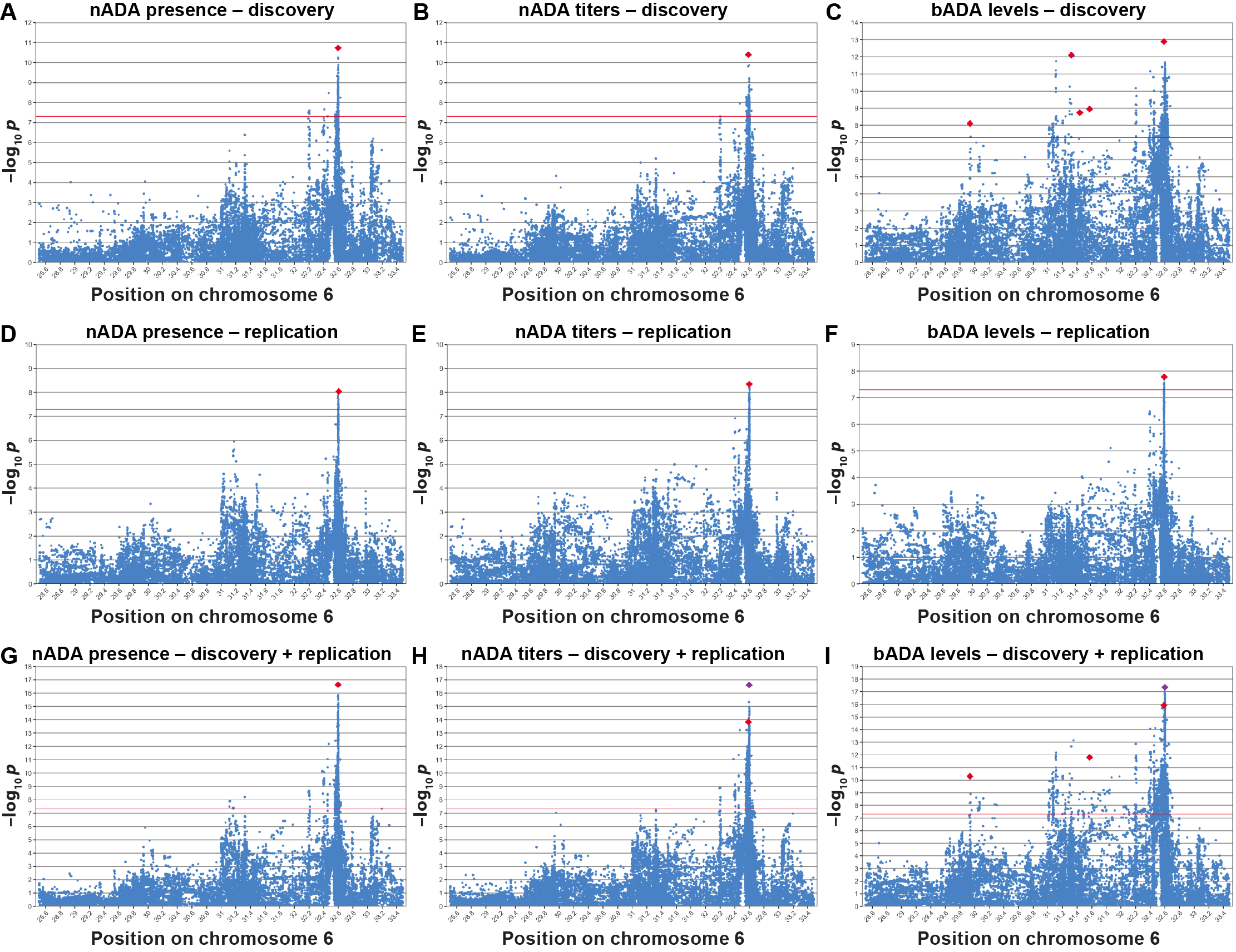

Supplement: Supplementary file 8 — Additional file 8. Manhattan plots of the MHC region of the GWAS across IFNβ preparations. Manhattan plots of the (A-C) discovery-stage, (D-F) replication-stage, and (G-I) pooled discovery + replication GWAS, showing only the MHC region. The red line between -log10p = 7 and -log10p = 8 indicates genome-wide significance. For (A-C) discovery-stage plots, the prioritized variants are labeled with red diamonds for (D-F) replication-stage plots, the top genome-wide significant variant is labeled with a red diamond, and for (G-I) pooled discovery + replication plots, the replicated variants are labeled with red diamonds, and the top pooled variant is labeled in magenta. [file 12916_2020_1769_MOESM8_ESM.png]

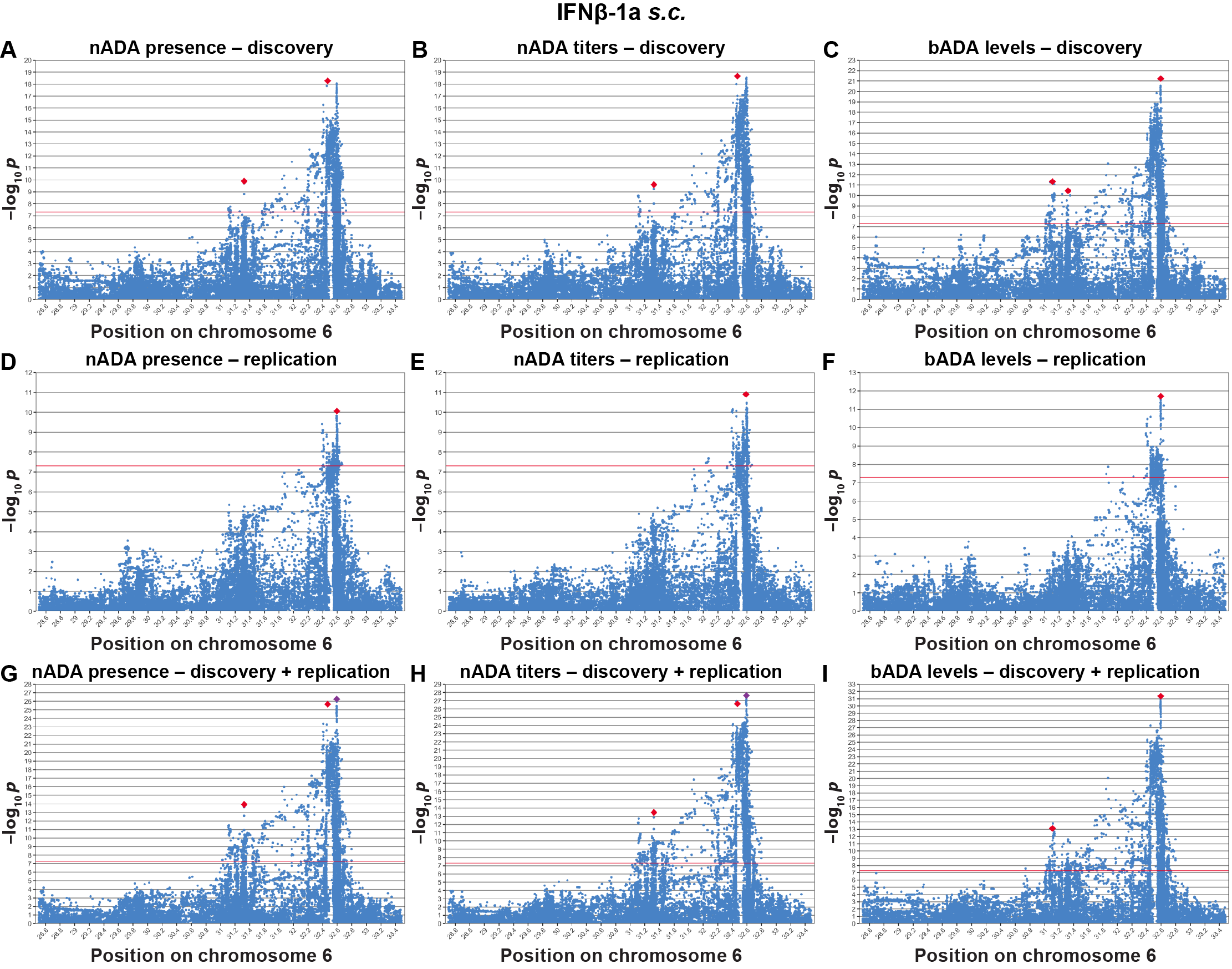

Supplement: Supplementary file 9 — Additional file 9. Manhattan plots of the MHC region of the GWAS on patients treated with IFNβ-1a s.c. Manhattan plots of the (A-C) discovery-stage, (D-F) replication-stage, and (G-I) pooled discovery + replication GWAS, showing only the MHC region. The red line between -log10p = 7 and -log10p = 8 indicates genome-wide significance. For (A-C) discovery-stage plots, the prioritized variants are labeled with red diamonds, for (D-F) replication-stage plots, the top genome-wide significant variant is labeled with a red diamond, and for (G-I) pooled discovery + replication plots, the replicated variants are labeled with red diamonds, and the top variant from the pooled analysis is labeled in magenta. [file 12916_2020_1769_MOESM9_ESM.png]

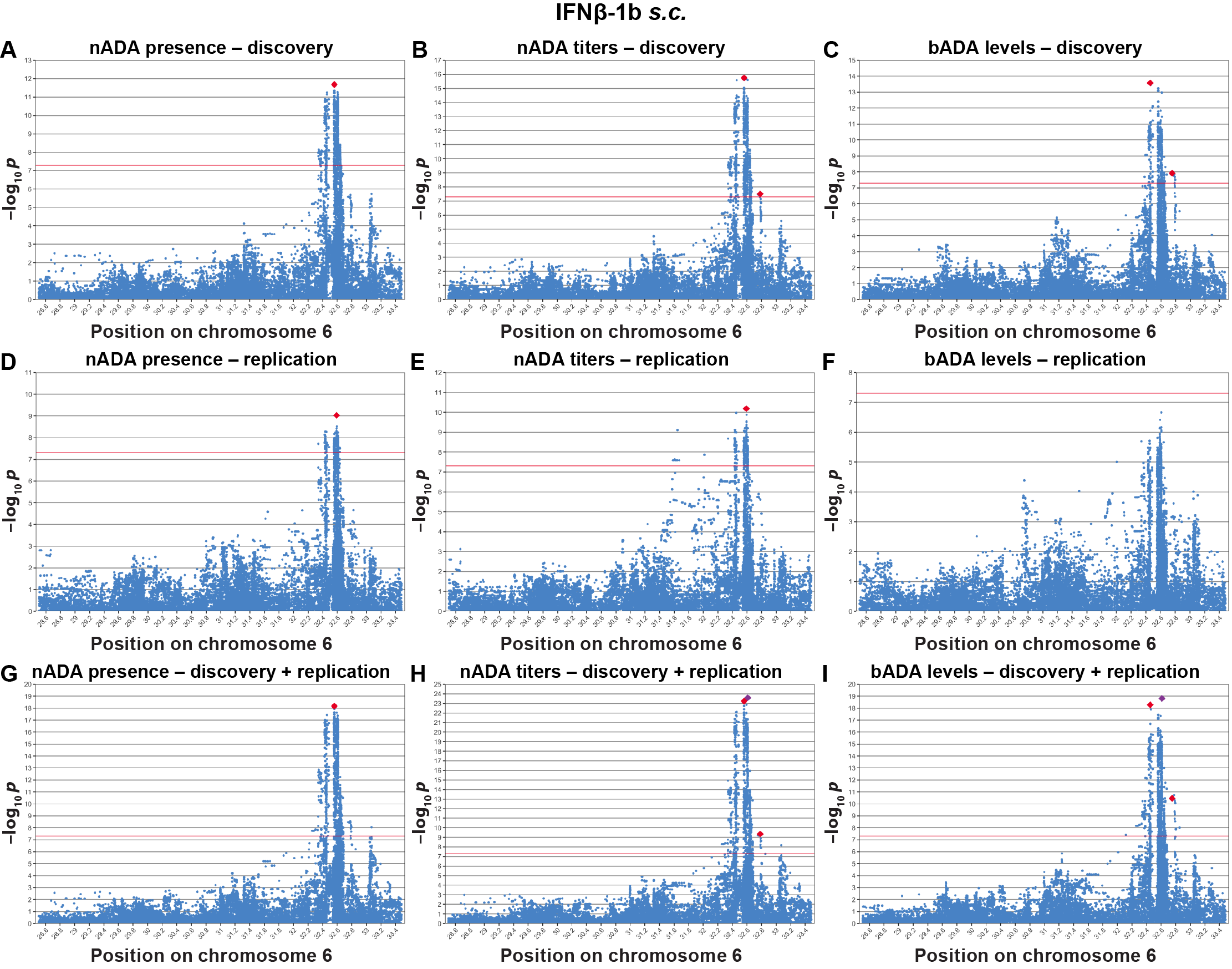

Supplement: Supplementary file 10 — Additional file 10. Manhattan plots of the MHC region of the GWAS on patients treated with IFNβ-1b s.c. Manhattan plots of the (A-C) discovery-stage, (D-F) replication-stage, and (G-I) pooled discovery + replication GWAS, showing only the MHC region. The red line between -log10p = 7 and -log10p = 8 indicates genome-wide significance. For (A-C) discovery-stage plots, the prioritized variants are labeled with red diamonds, for (D-F) replication-stage plots, the top genome-wide significant variant is labeled with a red diamond, and for (G-I) pooled discovery + replication plots, the replicated variants are labeled with red diamonds, and the top variant from the pooled analysis is labeled in magenta. [file 12916_2020_1769_MOESM10_ESM.png]

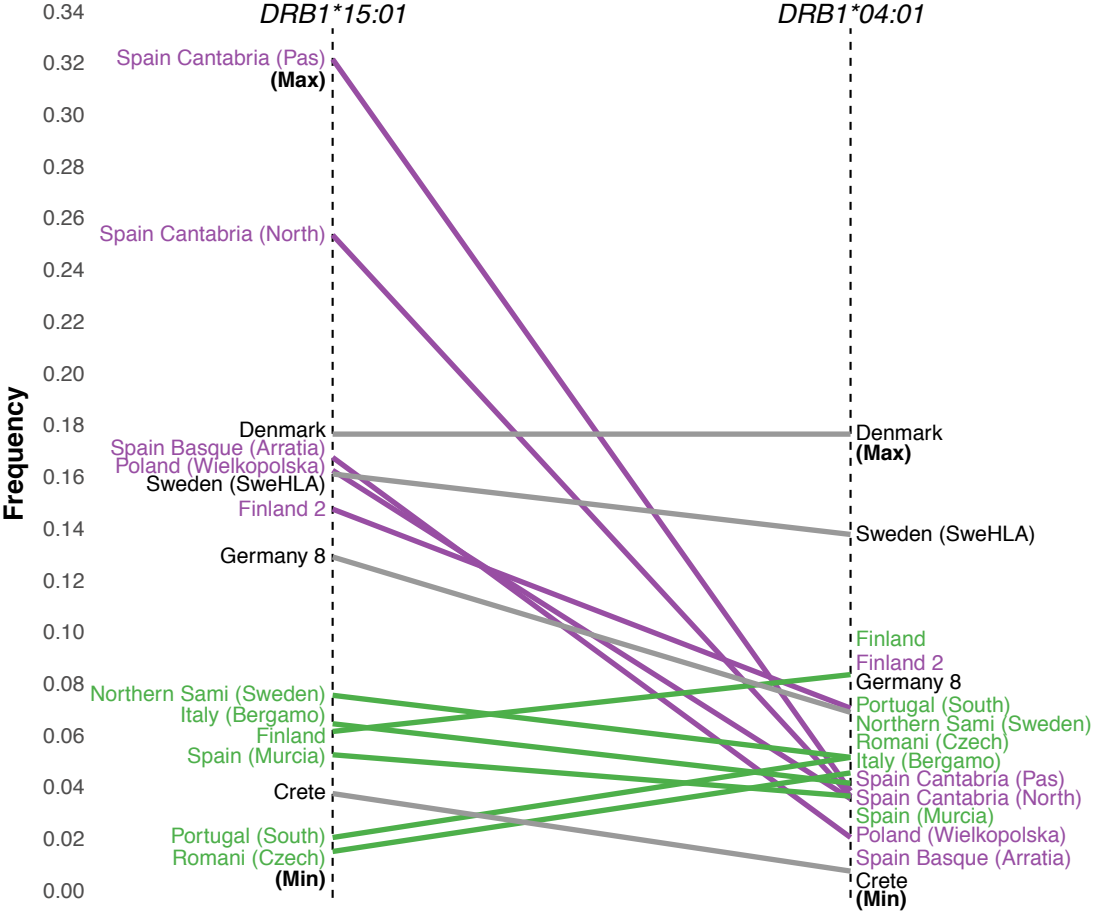

Supplement: Supplementary file 27 — Additional file 27. Comparison of allele frequencies for HLA-DRB1*15:01 and HLA-DRB1*04:01. The allele frequencies (AF) were queried from allelefrequencies.net on July 27th 2020 [48]. All four-digit European Silver and Gold populations with data on both HLA-DRB1*15:01 and HLA-DRB1*04:01 were used and populations with relative differences for both alleles are shown (i.e., with an AF above or below the average for one allele without the other allele being in the same group). Populations with an AF below the average for HLA-DRB1*15:01 and above the average for HLA-DRB1*04:01 are colored in green. Populations with an AF above the average for HLA-DRB1*15:01 and below the average for HLA-DRB1*04:01 are colored in magenta. In addition, the European populations with the highest or lowest AF for the respective allele (if not already present) as well as the largest German population and the Swedish SweHLA sample [47] are shown in gray. [file 12916_2020_1769_MOESM27_ESM.pdf]
