# Supplementary material for: Treatment- and population-specific genetic risk factors for anti-drug antibodies against interferon-beta: a GWAS
Source: BMC Med. 2020 Nov 4;18:298. doi: 10.1186/s12916-020-01769-6 (PMC7641861; doi:10.1186/s12916-020-01769-6)

Regional association plots of replicated and top pooled GWAS variants in the analysis across treatment preparations.

Regional association plots of variants from the GWAS generated using LocusZoom v1.4 and the 1000 Genomes 1000G_Nov2014 EUR reference panel. The color of dots indicates LD with the lead variant (pink). Gray dots represent signals with missing LD r^2 values. If no LD information was present in the database on the top variant, LD with the variant showing the second-lowest *p*-value is indicated. The grey line indicates genome-wide significance. cM: centimorgan, chr: chromosome, Mb: mega base pairs.

Regional association plot for variant rs9281971 in the analysis of **nADA presence** in the **discovery-stage** GWAS of combined treatments. LD information is shown for variant chr6:32597924 instead of rs9281971.

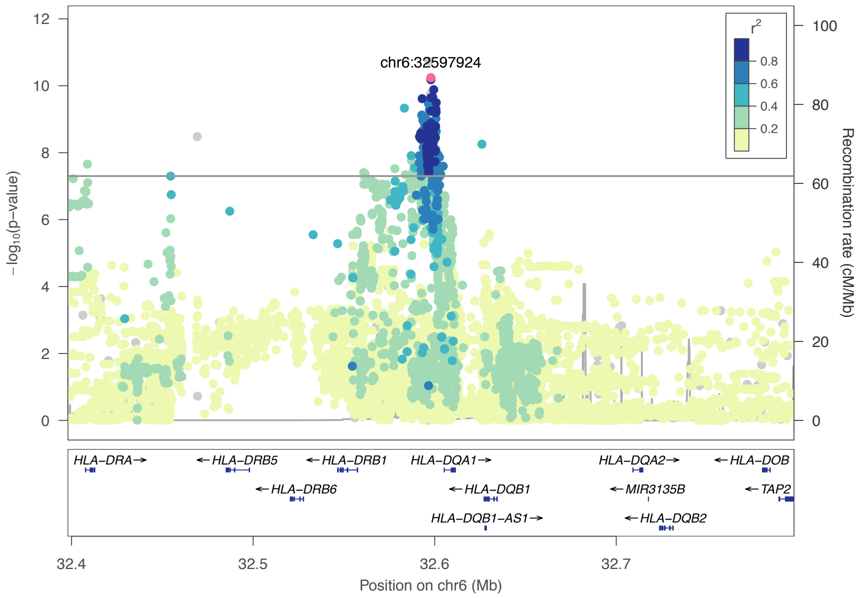

Regional association plot for variant rs9281971 in the analysis of **nADA presence** in the pooled **discovery + replication** GWAS of combined treatments. LD information is shown for variant chr6:32597924 instead of rs9281971.

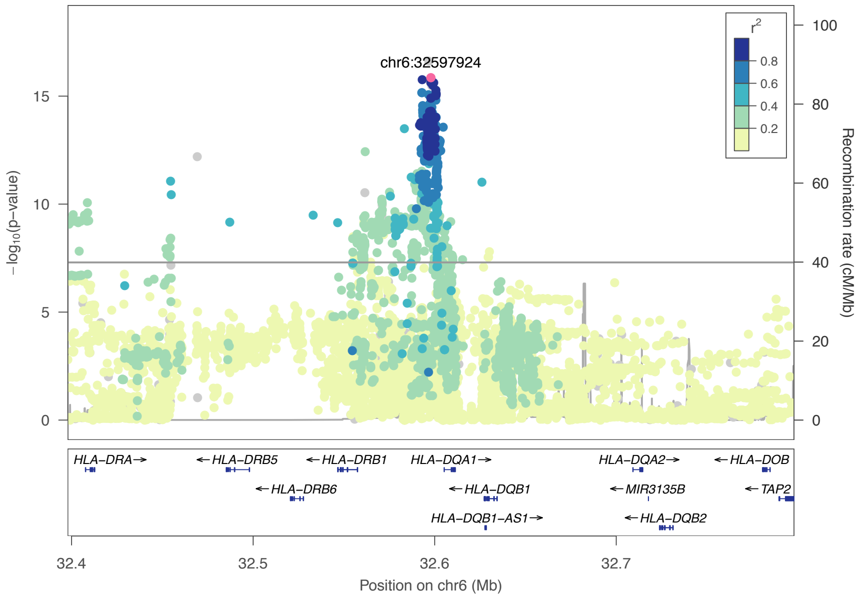

Regional association plot for variant rs9271377 in the analysis of **nADA titers** in the **discovery-stage** GWAS of combined treatments.

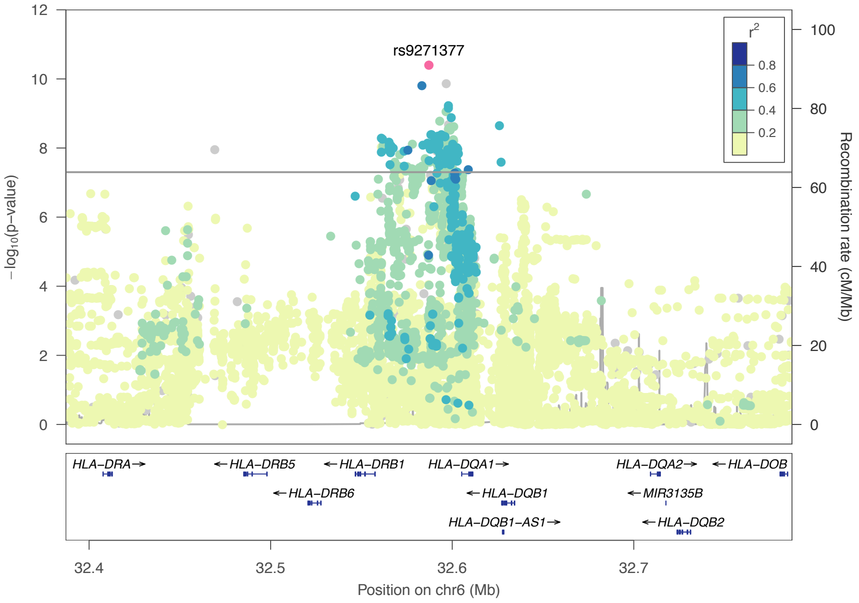

Regional association plot for variant rs9281971 in the analysis of **nADA titers** in the pooled **discovery + replication** GWAS of combined treatments. LD information is shown for variant chr6:32593179 instead of rs9281971.

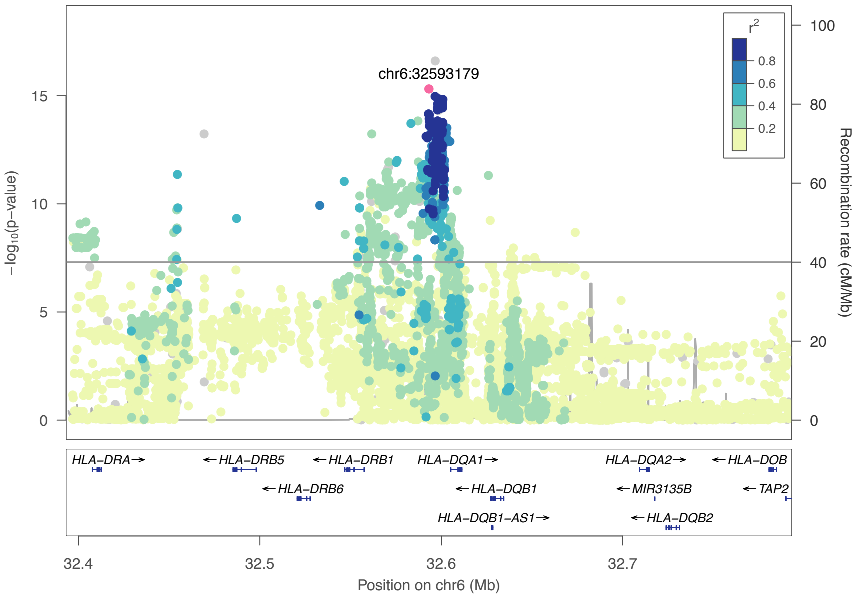

Regional association plot for variant rs9271377 in the analysis of **bADA levels** in in the **discovery-stage** GWAS of combined treatments.

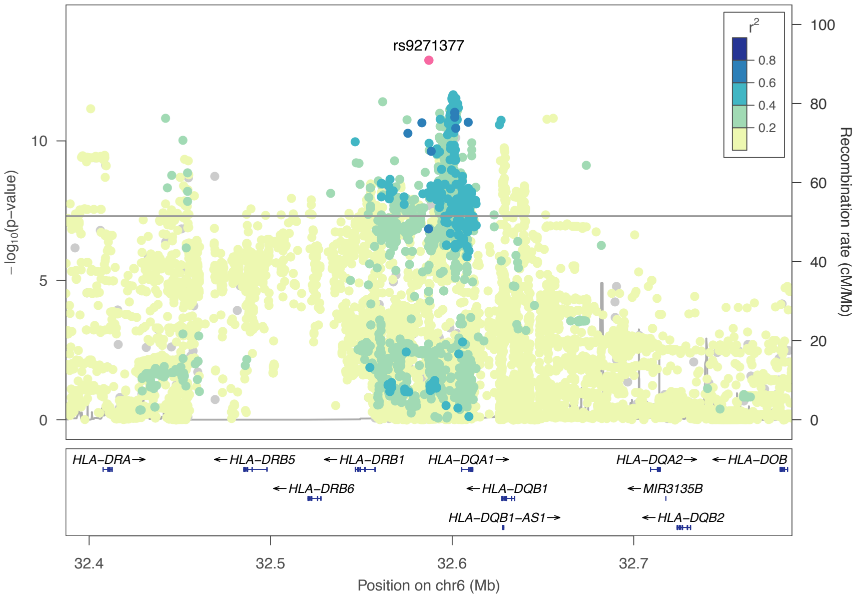

Regional association plot for variant rs9267515 in the analysis of **bADA levels** in in the **discovery-stage** GWAS of combined treatments.

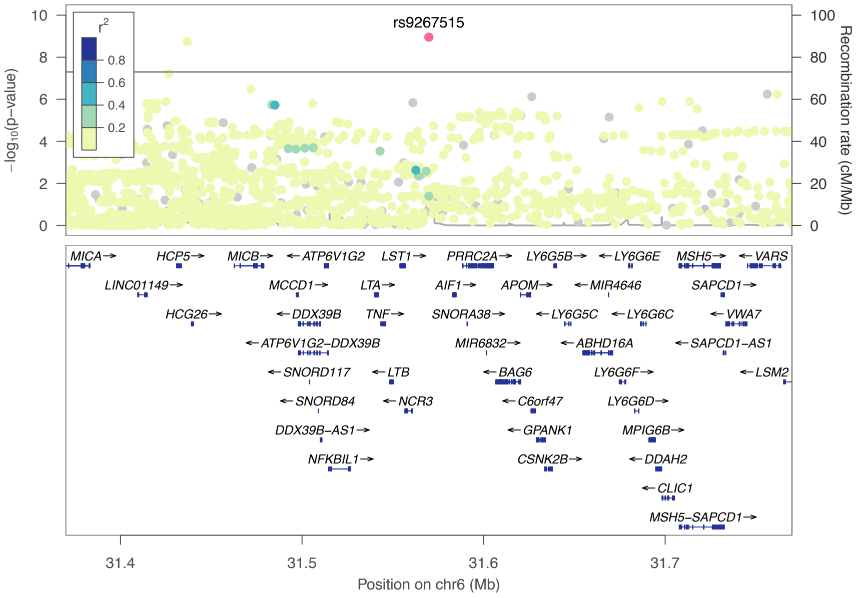

Regional association plot for variant rs9260765in the analysis of **bADA levels** in in the **discovery-stage** GWAS of combined treatments.

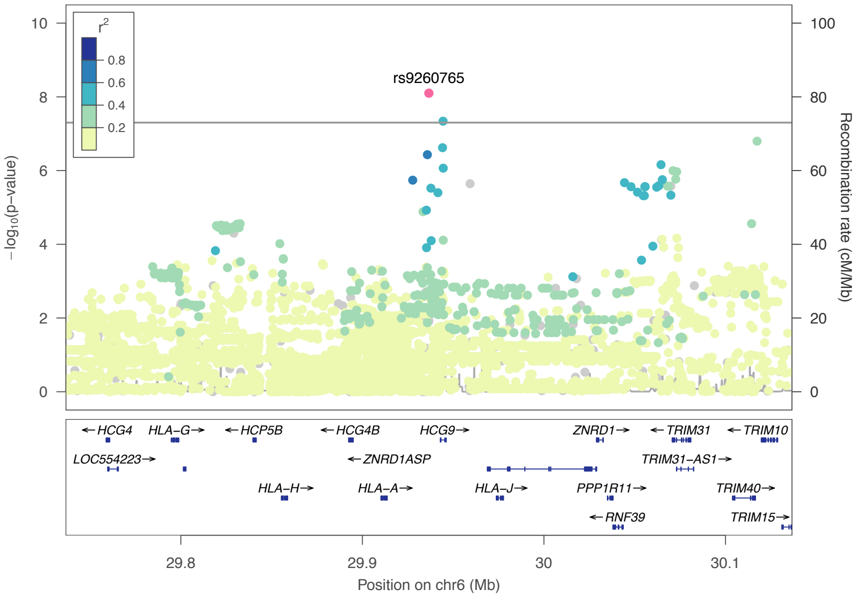

Regional association plot for variant rs9272071in the analysis of **bADA levels** in in the pooled **discovery + replication** GWAS of combined treatments.

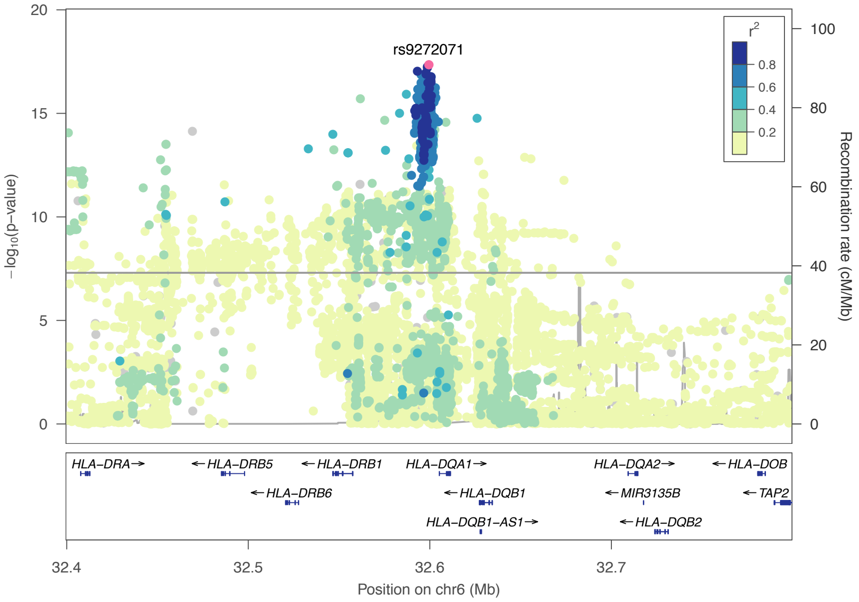

Supplement: Supplementary file 13 — Additional file 13. Regional association plots of the top GWAS variants in the analysis across IFNβ preparations. Regional association plots of variants from the GWAS generated using LocusZoom v1.4 and the 1000 Genomes 1000G_Nov2014 EUR reference panel [72]. The color of dots indicates LD with the lead variant (pink). Gray dots represent signals with missing LD r2 values. If no LD information was present in the database on the top variant, LD with the variant showing the second-lowest p-value is indicated. The gray line indicates genome-wide significance. cM: centimorgan, chr: chromosome, Mb: mega base pairs. [file 12916_2020_1769_MOESM13_ESM.pdf]
