# Supplementary material for: Treatment- and population-specific genetic risk factors for anti-drug antibodies against interferon-beta: a GWAS
Source: BMC Med. 2020 Nov 4;18:298. doi: 10.1186/s12916-020-01769-6 (PMC7641861; doi:10.1186/s12916-020-01769-6)

Forest plots of replicated and top pooled GWAS variants and *HLA* alleles in the analysis across treatment preparations.

Green: IFNβ-1a *s.c.*, blue: IFNβ-1a *i.m.*, orange: IFNβ-1b *s.c.*, magenta: pooled discovery-/replication-stage analyses. D. = discovery, R. = replication, P. = pooled discovery + replication.

Forest plot for the GWAS variant rs9281971 in the combined analysis of **nADA presence**.

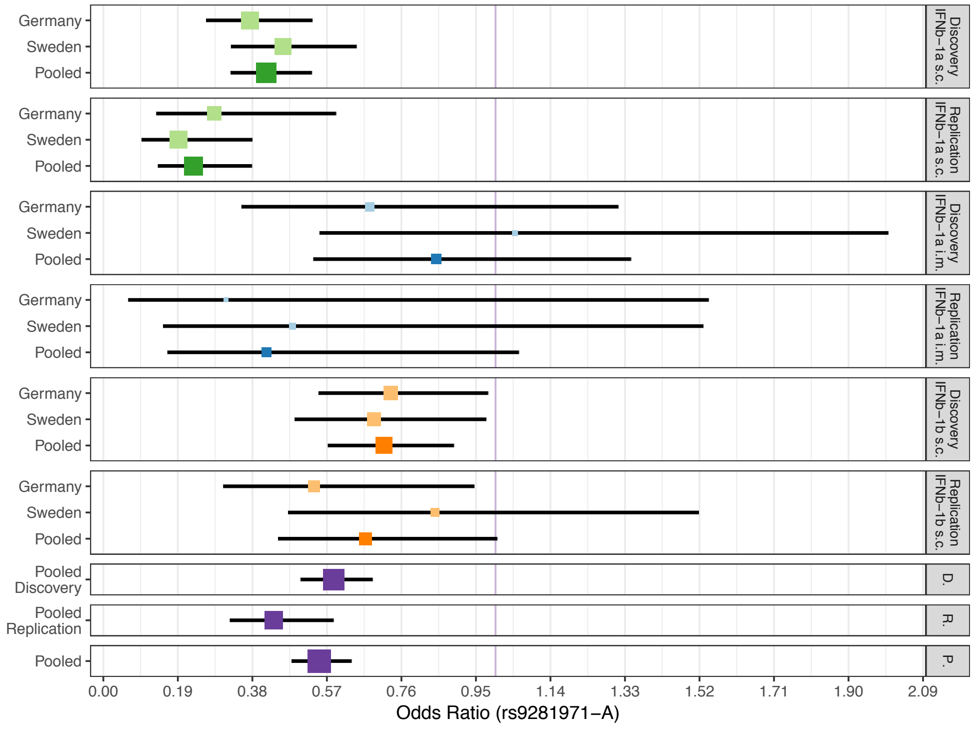

Forest plot for the GWAS variant rs9271377 in the combined analysis of **nADA titers**.

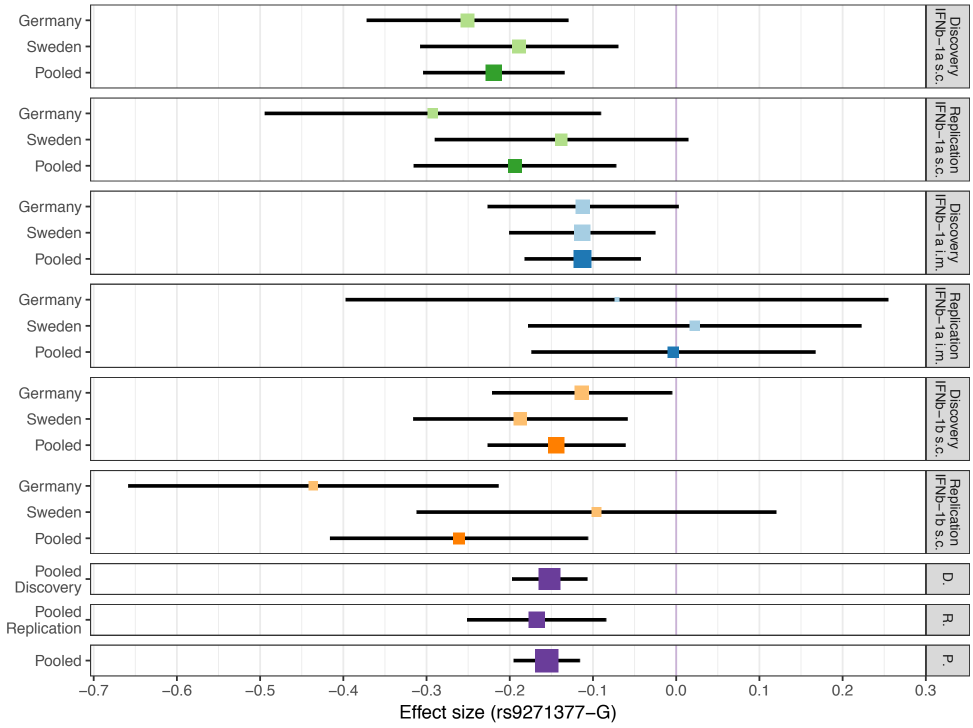

Genetic risk for anti-drug antibodies against interferon-beta – **Forest plots**

Forest plot for the GWAS variant rs9281971 in the combined analysis of **nADA titers**.

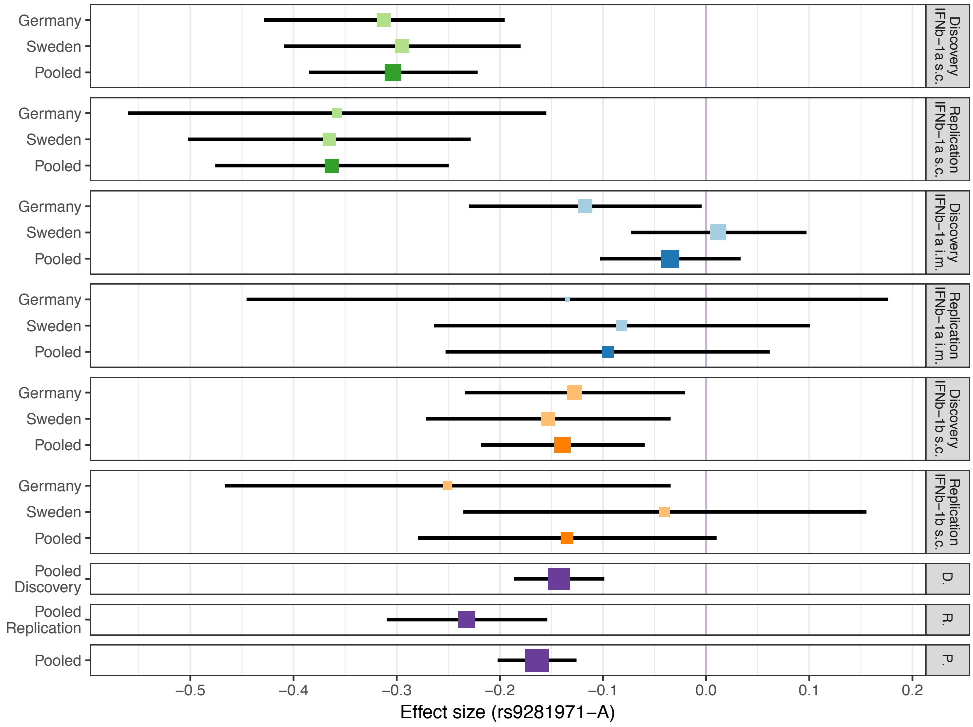

Forest plot for the GWAS variant rs9271377 in the combined analysis of **bADA levels**.

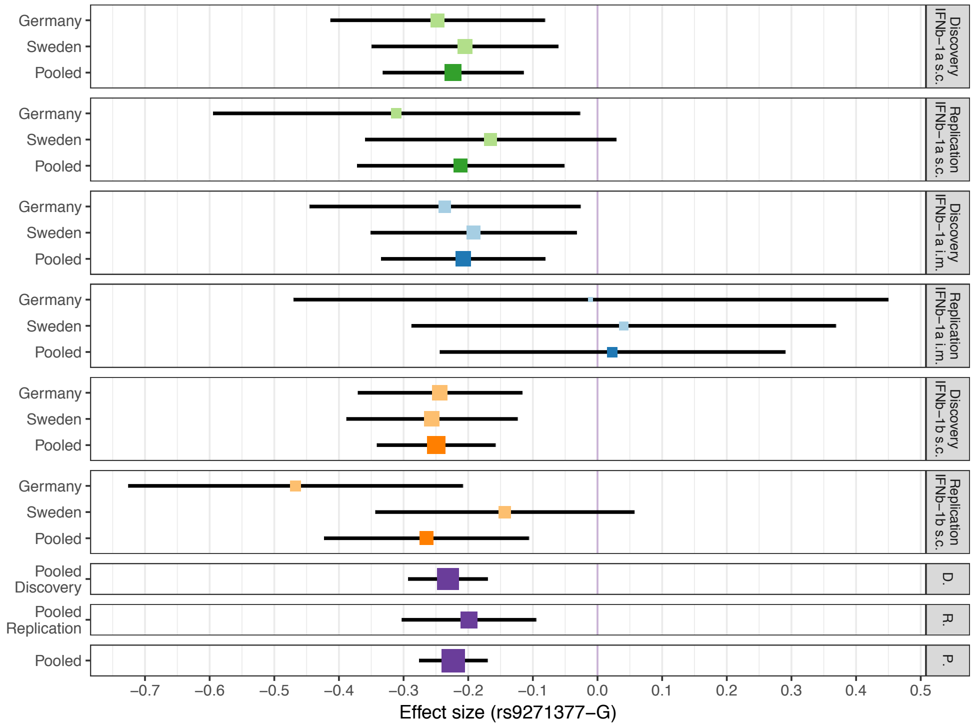

Forest plot for the GWAS variant rs9267515 in the combined analysis of **bADA levels**.

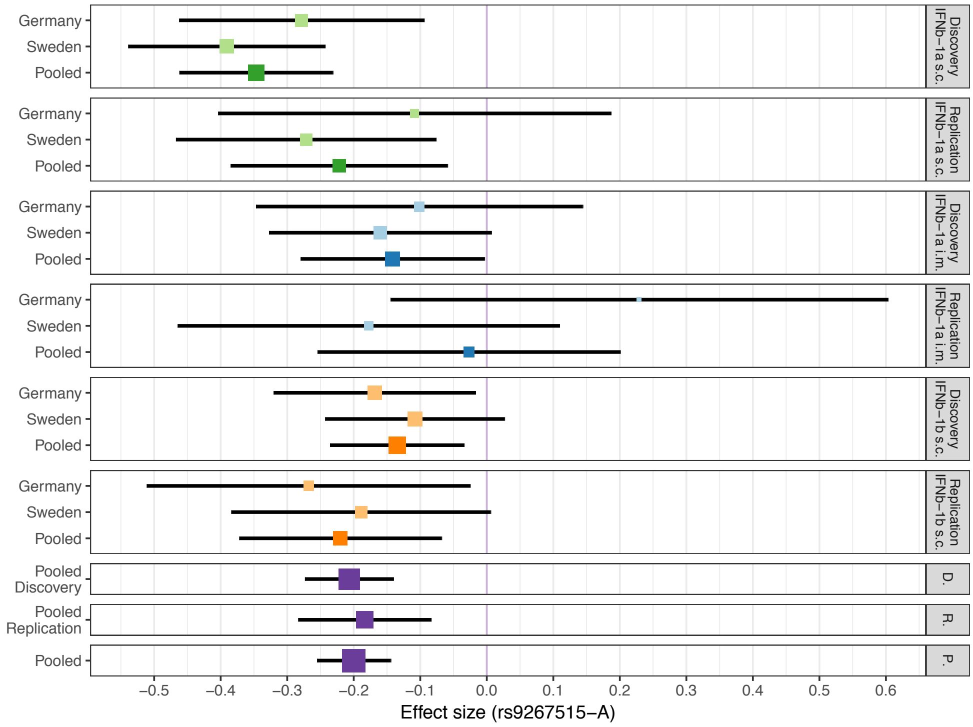

Forest plot for the GWAS variant rs9260765 in the combined analysis of **bADA levels**.

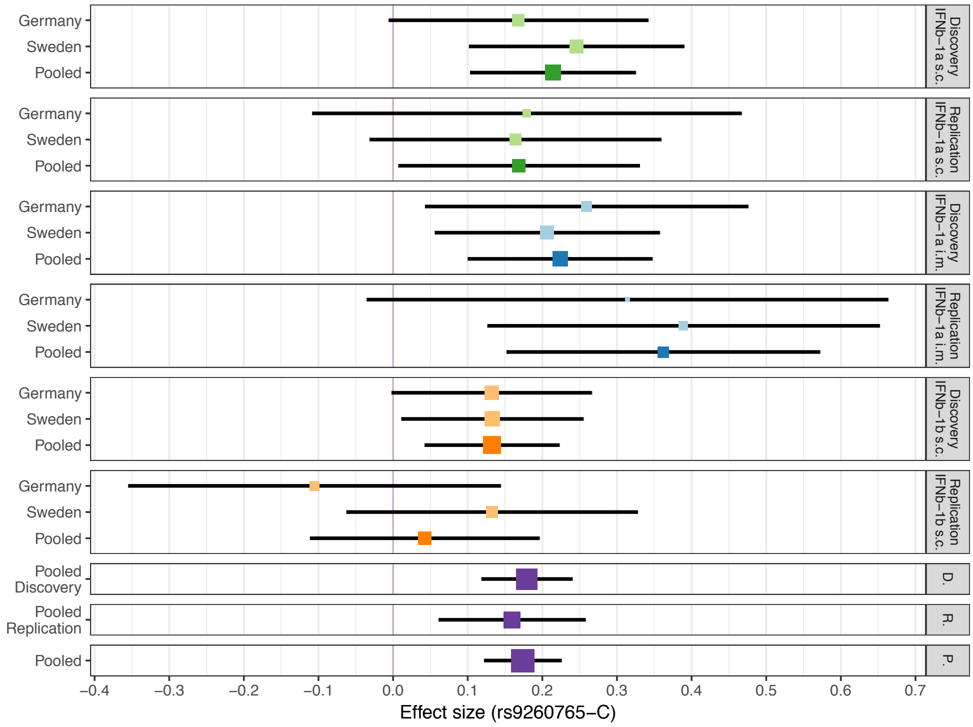

Forest plot for the GWAS variant rs9272071 in the combined analysis of **bADA levels**.

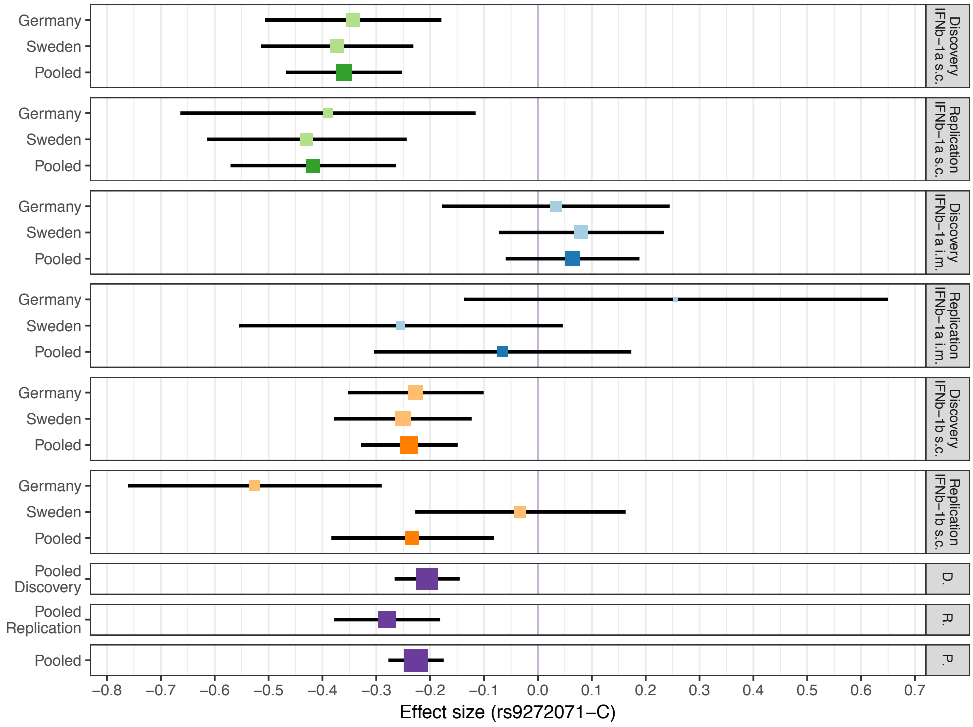

Forest plot for the *HLA* allele *HLA-DQA1*01:02* in the combined analysis of **bADA levels**.

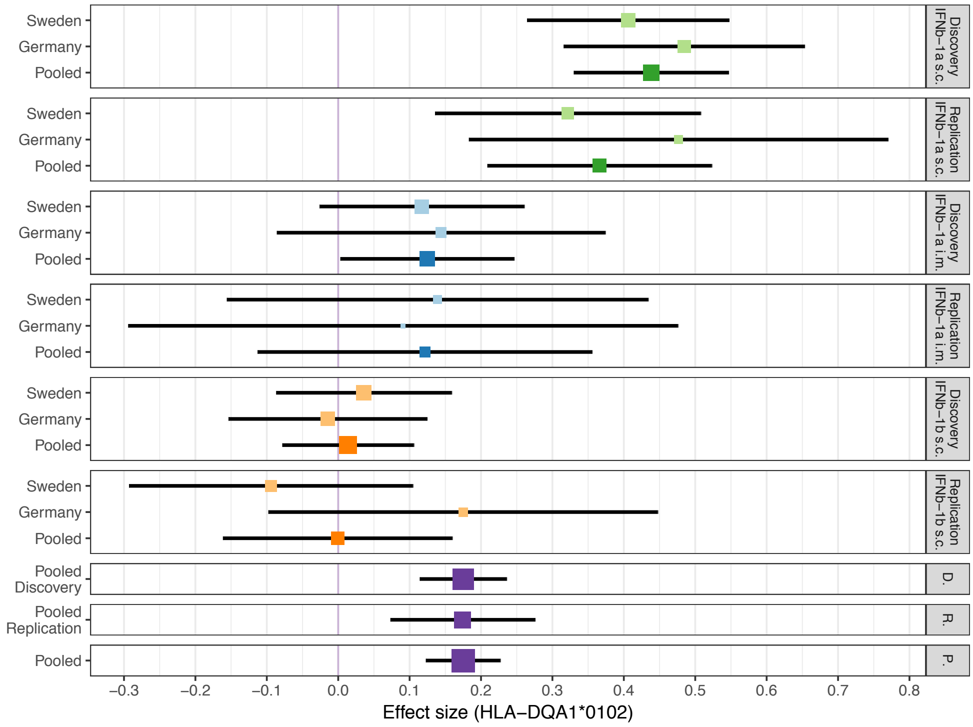

Forest plot for the extended *HLA* haplotype *B7-DQ6* in the combined analysis of **bADA levels**.

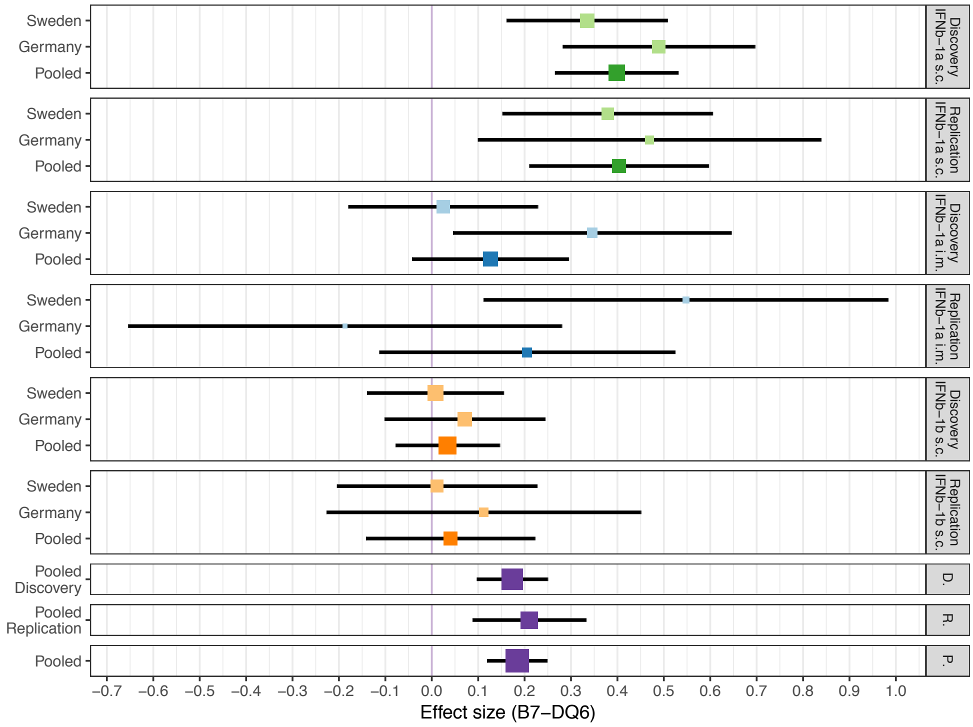

Forest plot for the extended *HLA* haplotype *DR3-DQ2* in the combined analysis of **bADA levels**.

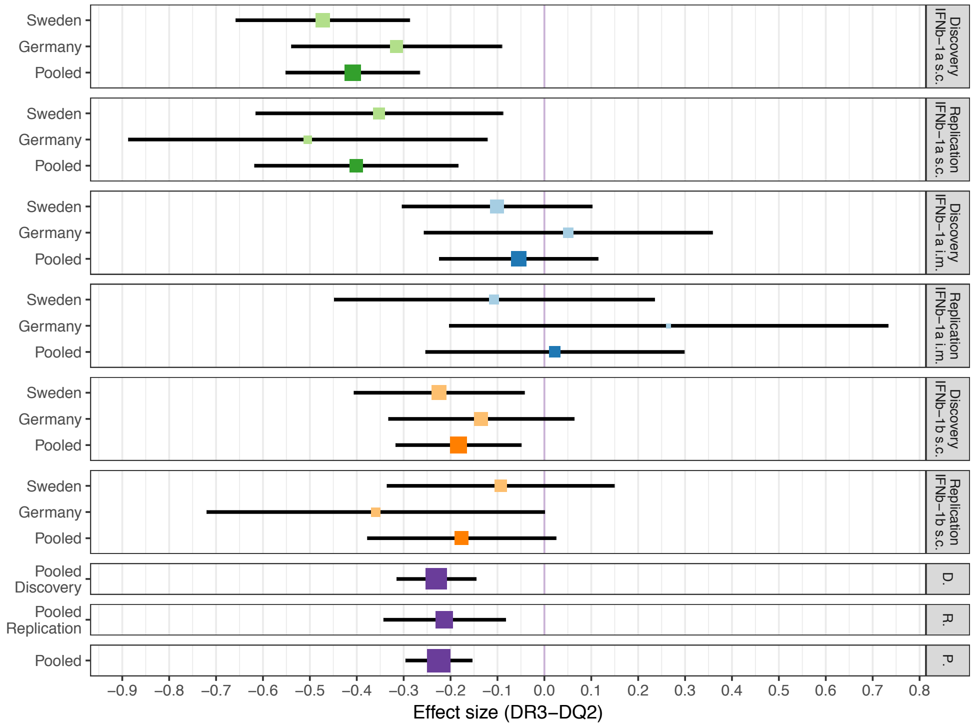

Supplement: Supplementary file 14 — Additional file 14. Forest plots of the top GWAS variants and HLA alleles in the analysis across IFNβ preparations. Green: IFNβ-1a s.c., blue: IFNβ-1a i.m., orange: IFNβ-1b s.c., magenta: pooled discovery−/replication-stage analyses. D. = discovery, R. = replication, P. = pooled discovery + replication. [file 12916_2020_1769_MOESM14_ESM.pdf]
