# Supplementary material for: Treatment- and population-specific genetic risk factors for anti-drug antibodies against interferon-beta: a GWAS
Source: BMC Med. 2020 Nov 4;18:298. doi: 10.1186/s12916-020-01769-6 (PMC7641861; doi:10.1186/s12916-020-01769-6)

Regional association plots of replicated and top pooled GWAS variants in the analysis of IFNβ-1a *s.c.*-treated patients.

Regional association plots of variants from the GWAS generated using LocusZoom v1.4 and the 1000 Genomes 1000G_Nov2014 EUR reference panel. The color of dots indicates LD with the lead variant (pink). Gray dots represent signals with missing LD r^2 values. If no LD information was present in the database on the top variant, LD with the variant showing the second-lowest *p*-value is indicated. The grey line indicates genome-wide significance. cM: centimorgan, chr: chromosome, Mb: mega base pairs.

Regional association plot for variant rs77278603 in the analysis of **nADA presence** in the **discovery-stage** GWAS of IFNβ-1a *s.c.*-treated patients.

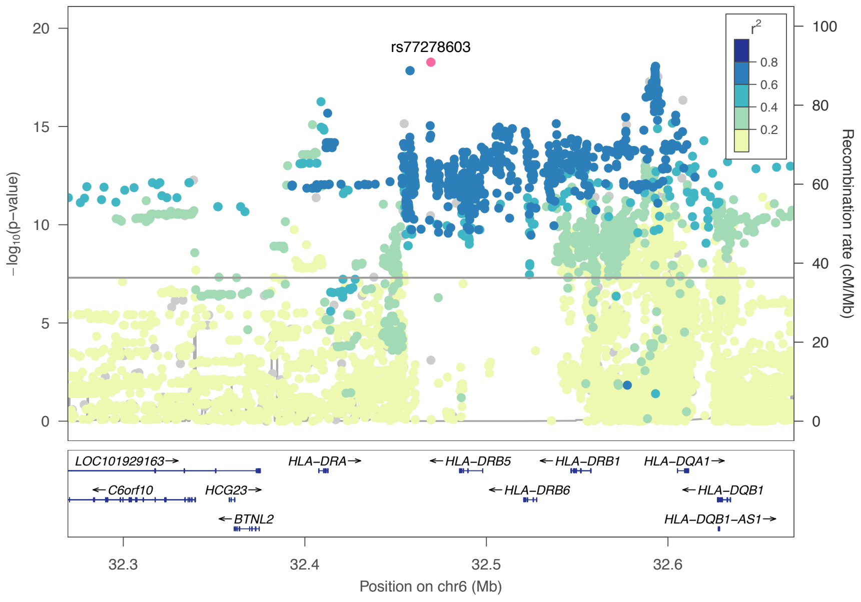

Regional association plot for variant rs1131204 in the analysis of **nADA presence** in the **discovery-stage** GWAS of IFNβ-1a *s.c.*-treated patients. LD information is shown for variant chr6:31324526 instead of rs1131204.

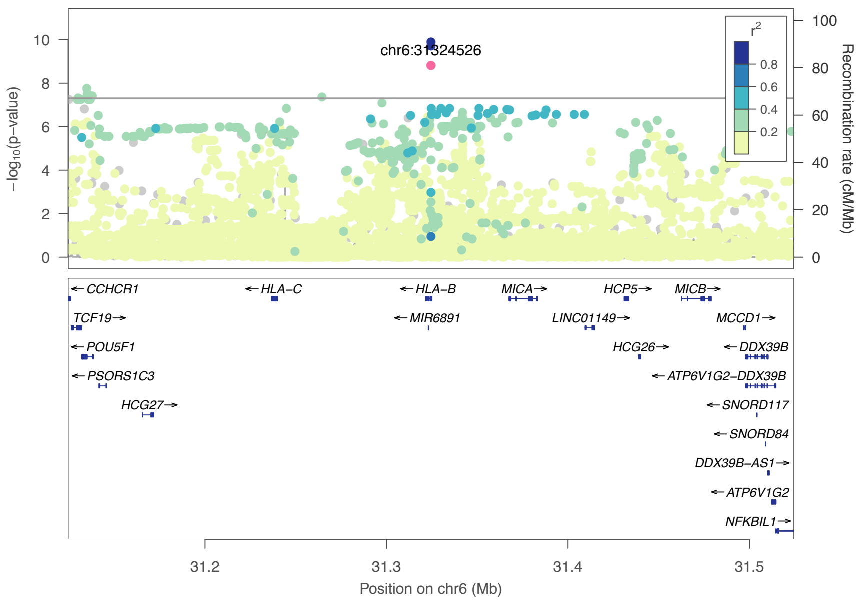

Regional association plot for variant rs9271700 in the analysis of **nADA presence** in the pooled **discovery + replication** GWAS of IFNβ-1a *s.c.*-treated patients.

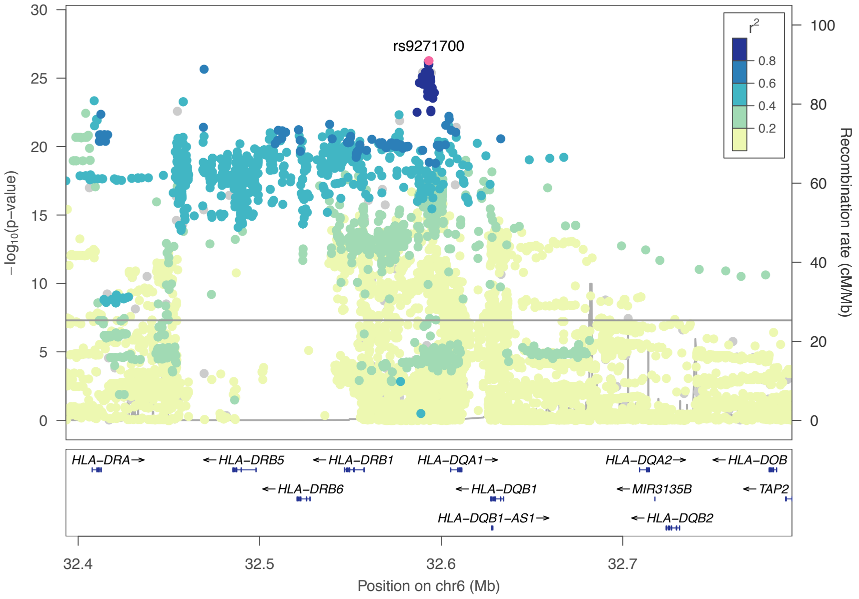

Regional association plot for variant rs77278603 in the analysis of **nADA titers** in the **discovery-stage** GWAS of IFNβ-1a *s.c.*-treated patients.

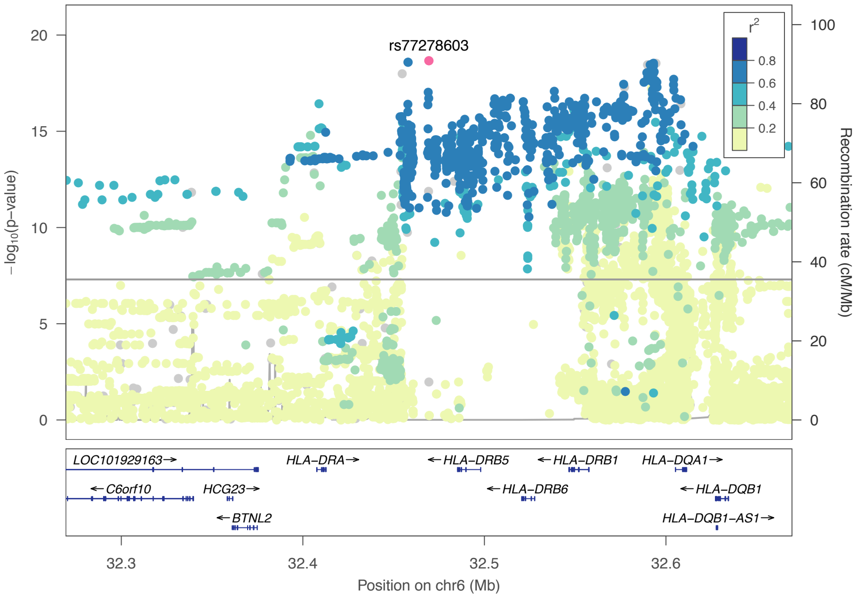

Regional association plot for variant rs1131204 in the analysis of **nADA titers** in the **discovery-stage** GWAS of IFNβ-1a *s.c.*-treated patients. LD information is shown for variant chr6:31324526 instead of rs1131204.

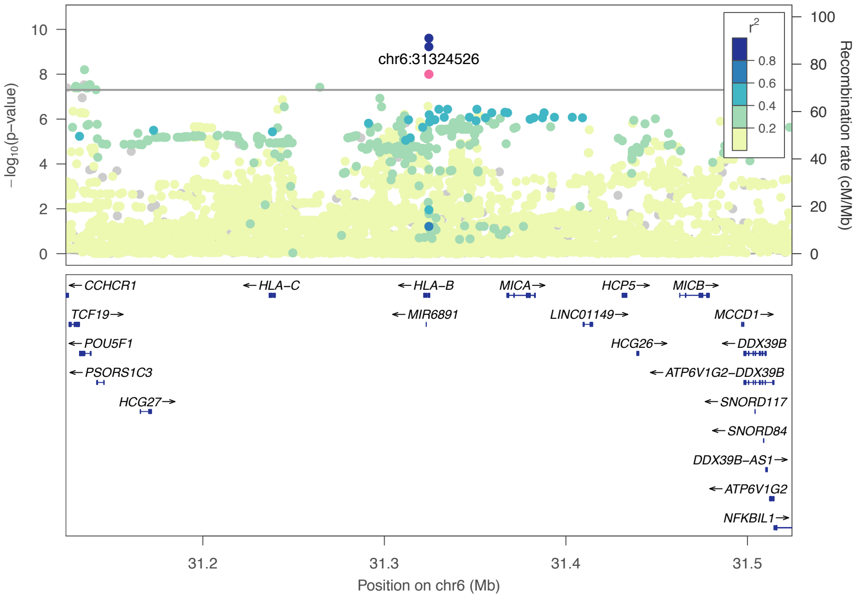

Regional association plot for variant rs9271673 in the analysis of **nADA titers** in the pooled **discovery + replication** GWAS of IFNβ-1a *s.c.*-treated patients.

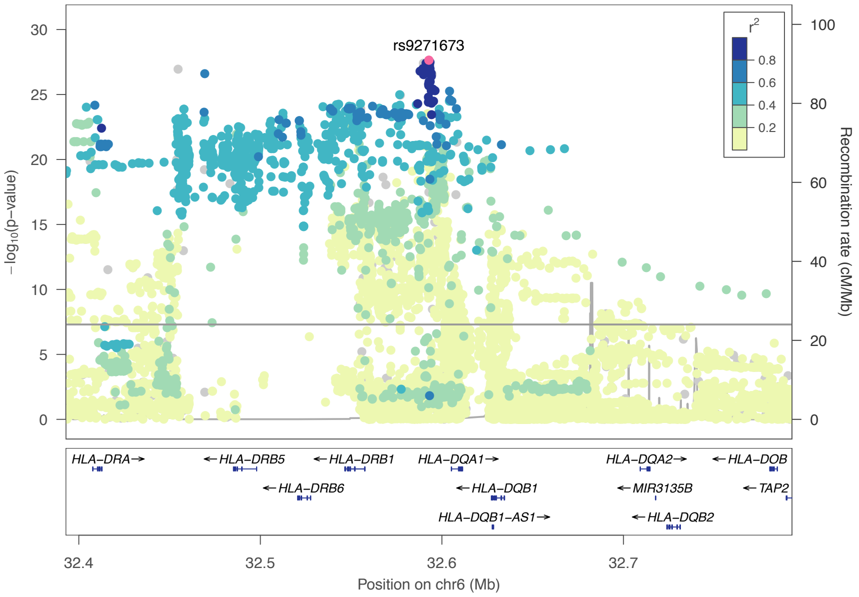

Regional association plot for variant rs9281962 in the analysis of **bADA levels** in the **discovery-stage** GWAS of IFNβ-1a *s.c.*-treated patients. LD information is shown for variant chr6:32592143 instead of rs9281962.

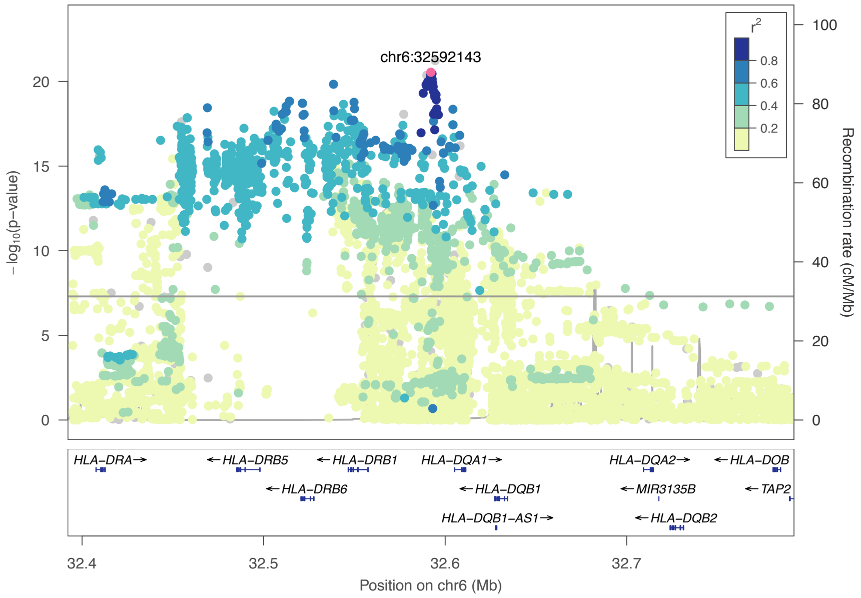

Regional association plot for variant rs130073 in the analysis of **bADA levels** in the **discovery-stage** GWAS of IFNβ-1a *s.c.*-treated patients.

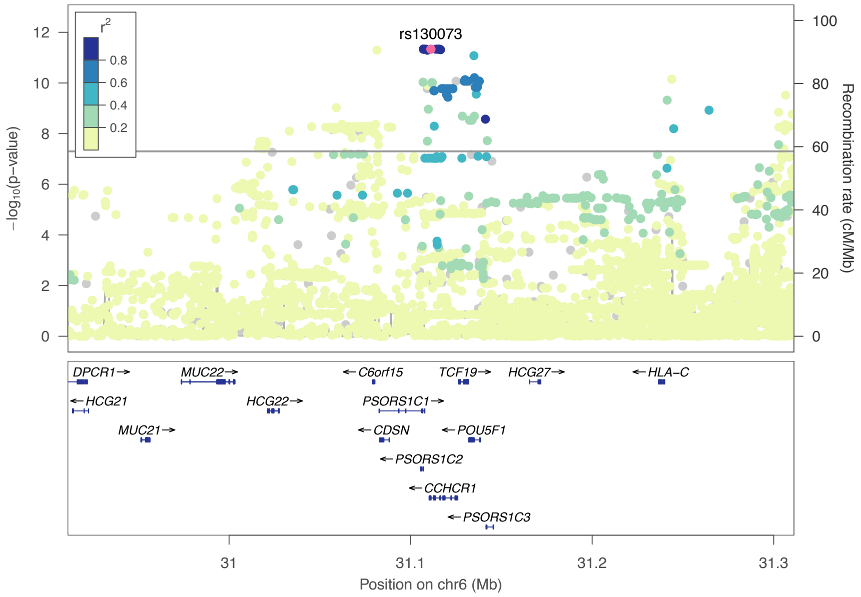

Regional association plot for variant rs9281962 in the analysis of **bADA levels** in the pooled **discovery + replication** GWAS of IFNβ-1a *s.c.*-treated patients. LD information is shown for variant chr6:32592143 instead of rs9281962.

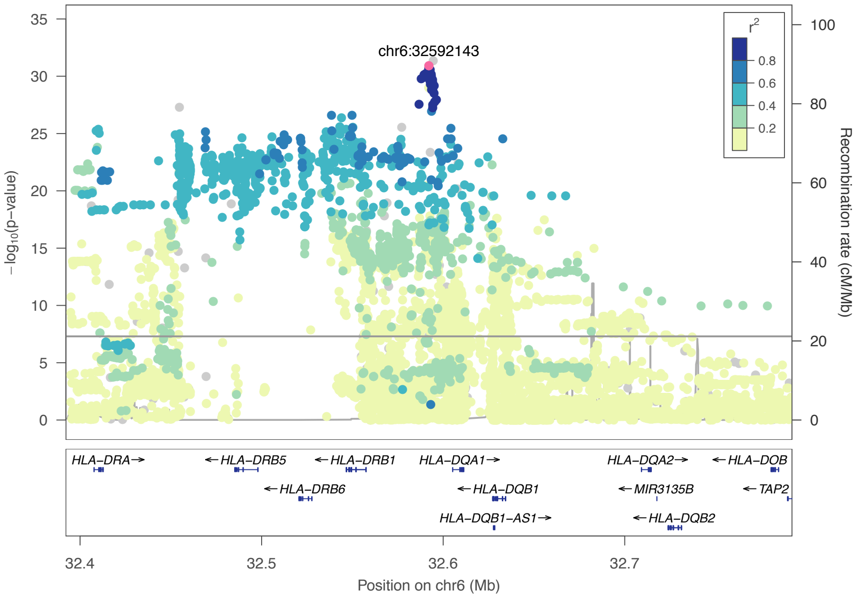

Supplement: Supplementary file 18 — Additional file 18. Regional association plots of the top GWAS variants in the analysis of IFNβ-1a s.c.-treated patients. Regional association plots of variants from the GWAS generated using LocusZoom v1.4 and the 1000 Genomes 1000G_Nov2014 EUR reference panel [72]. The color of dots indicates LD with the lead variant (pink). Gray dots represent signals with missing LD r2 values. If no LD information was present in the database on the top variant, LD with the variant showing the second-lowest p-value is indicated. The gray line indicates genome-wide significance. cM: centimorgan, chr: chromosome, Mb: mega base pairs. [file 12916_2020_1769_MOESM18_ESM.pdf]
