# Supplementary material for: Treatment- and population-specific genetic risk factors for anti-drug antibodies against interferon-beta: a GWAS
Source: BMC Med. 2020 Nov 4;18:298. doi: 10.1186/s12916-020-01769-6 (PMC7641861; doi:10.1186/s12916-020-01769-6)

Regional association plots of replicated and top pooled GWAS variants in the analysis of IFNβ-1b *s.c.*-treated patients.

Regional association plots of variants from the GWAS generated using LocusZoom v1.4 and the 1000 Genomes 1000G_Nov2014 EUR reference panel. The color of dots indicates LD with the lead variant (pink). Gray dots represent signals with missing LD r^2 values. If no LD information was present in the database on the top variant, LD with the variant showing the second-lowest *p*-value is indicated. The grey line indicates genome-wide significance. cM: centimorgan, chr: chromosome, Mb: mega base pairs.

Regional association plot for variant rs28366299 in the analysis of **nADA presence** in the **discovery-stage** GWAS of IFNβ-1b *s.c.*-treated patients.

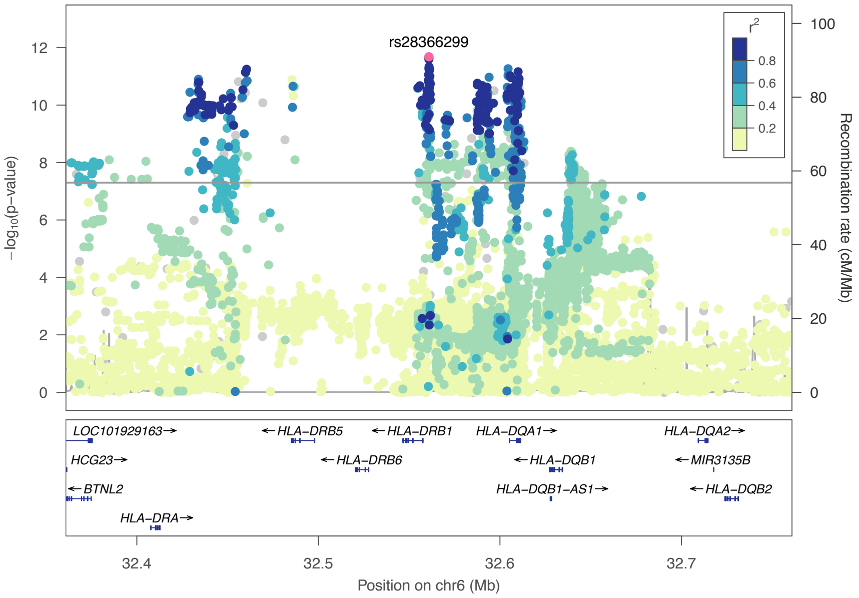

Regional association plot for variant rs28366299 in the analysis of **nADA presence** in the pooled **discovery + replication** GWAS of IFNβ-1b *s.c.*-treated patients.

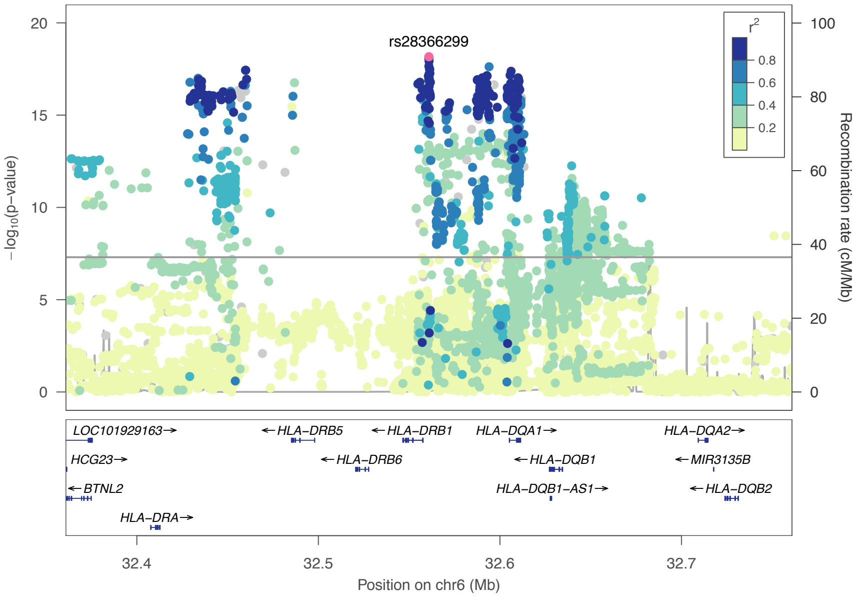

Regional association plot for variant rs28366299 in the analysis of **nADA titers** in the **discovery-stage** GWAS of IFNβ-1b *s.c.*-treated patients.

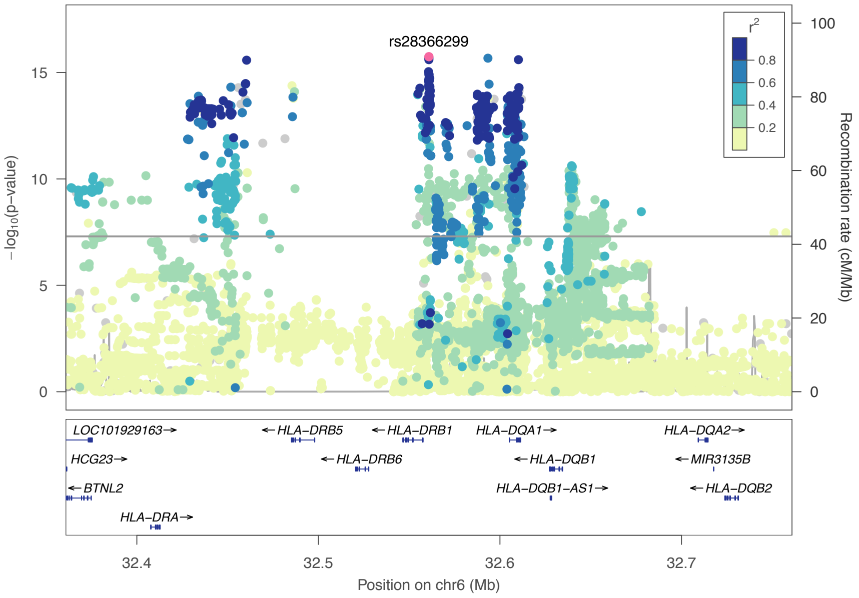

Regional association plot for variant rs2071479 in the analysis of **nADA titers** in the **discovery-stage** GWAS of IFNβ-1b *s.c.*-treated patients.

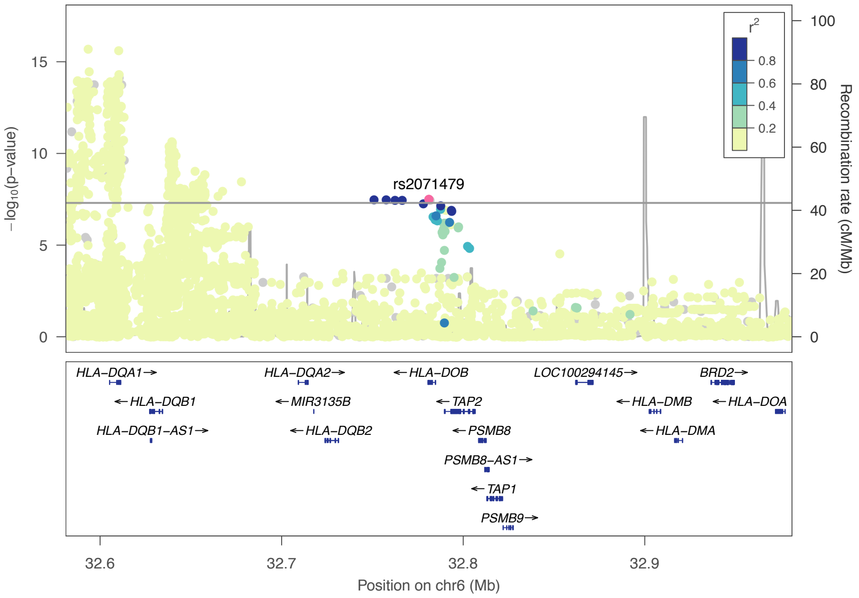

Regional association plot for variant rs9272775 in the analysis of **nADA titers** in the pooled **discovery + replication** GWAS of IFNβ-1b *s.c.*-treated patients.

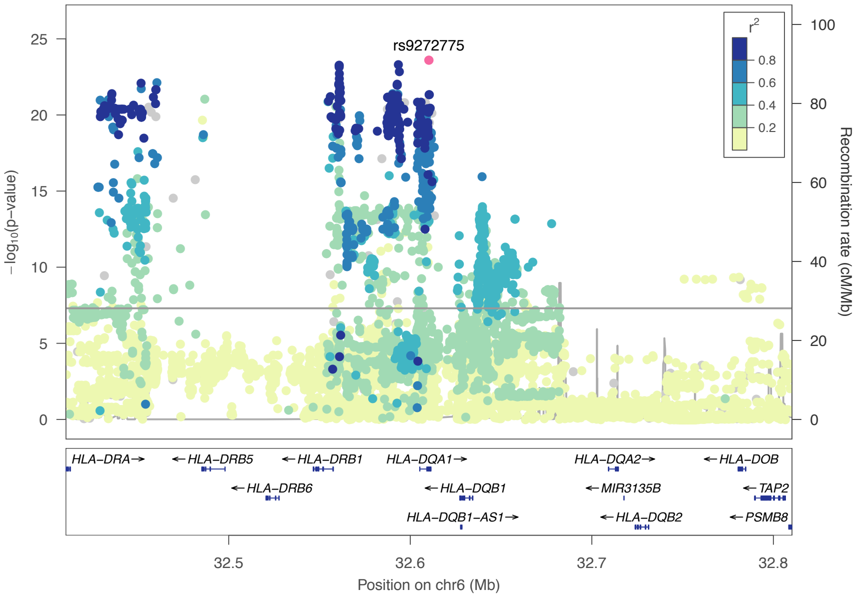

Regional association plot for variant rs78279385 in the analysis of **bADA levels** in the **discovery-stage** GWAS of IFNβ-1b *s.c.*-treated patients.

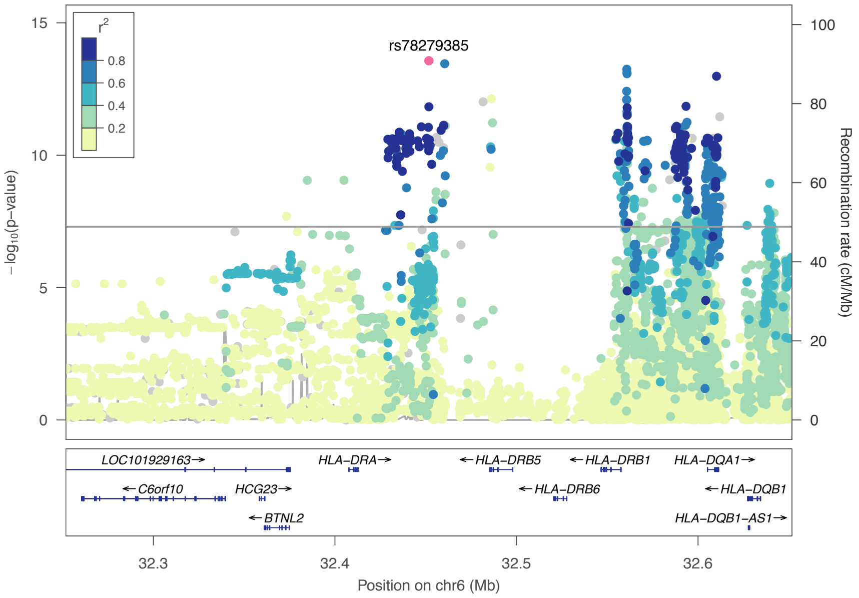

Regional association plot for variant rs57912571 in the analysis of **bADA levels** in the **discovery-stage** GWAS of IFNβ-1b *s.c.*-treated patients.

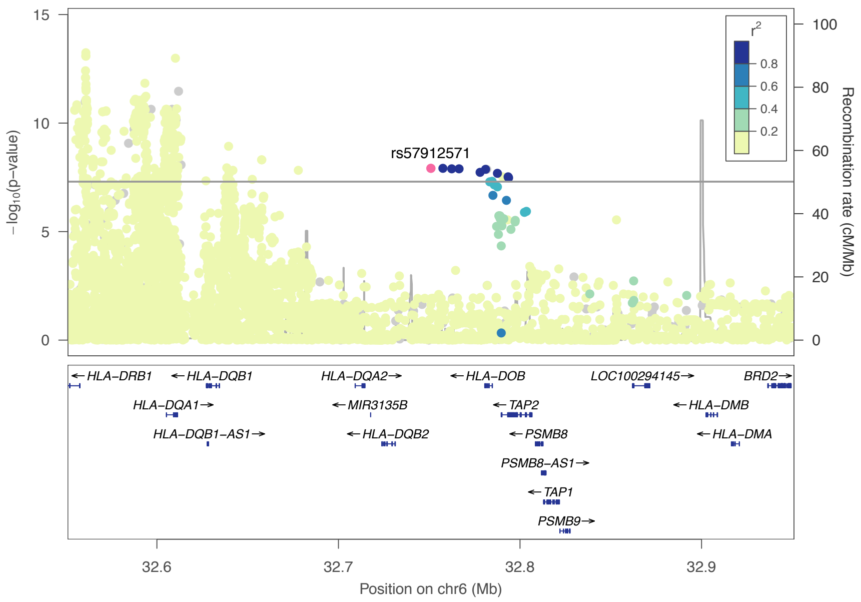

Regional association plot for variant rs9272775 in the analysis of **bADA levels** in the pooled **discovery + replication** GWAS of IFNβ-1b *s.c.*-treated patients.

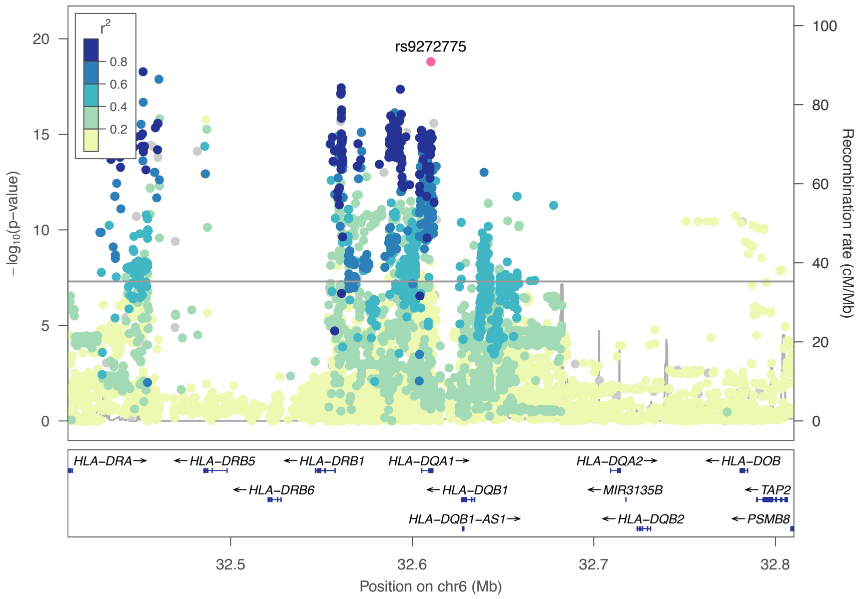

Regional association plot for variant rs559242105, conditioned for rs28366299, in the conditional analysis of **nADA presence** in the **discovery + replication** pooled dataset of IFNβ-1b *s.c.*-treated patients. LD information is shown for variant rs9277379 instead of rs559242105.

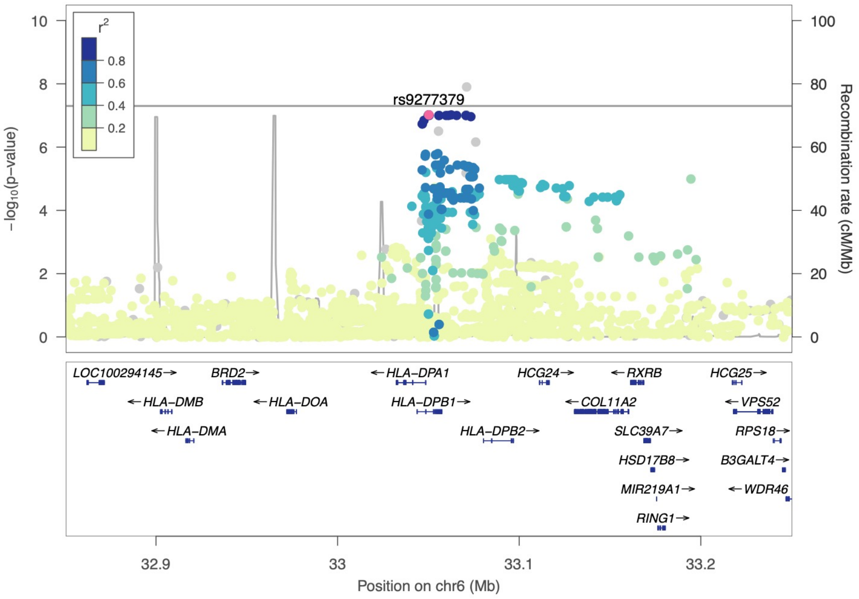

Regional association plot for variant rs559242105, conditioned for rs9272775, in the conditional analysis of **nADA titers** in the **discovery + replication** pooled dataset of IFNβ-1b *s.c.*-treated patients. LD information is shown for variant rs9277379 instead of rs559242105.

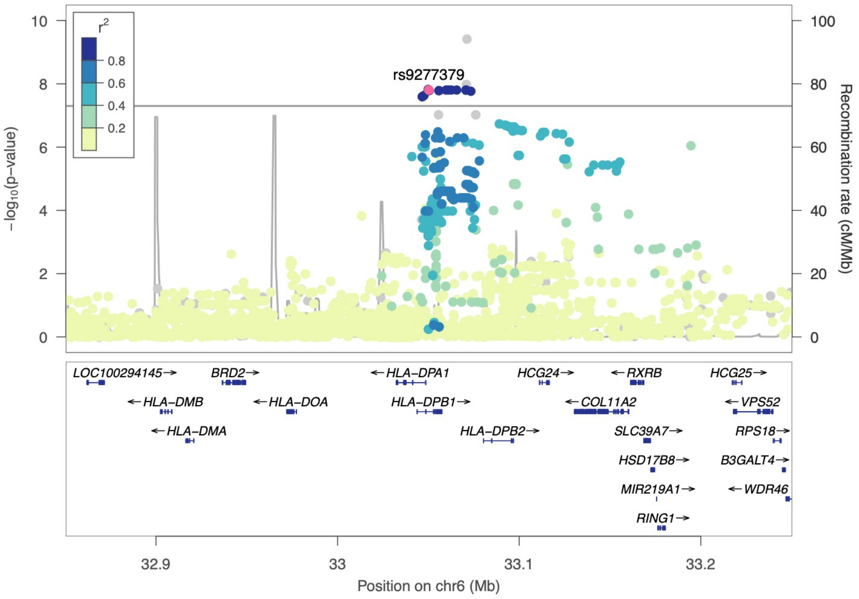

Regional association plot for variant rs17205731, conditioned for *HLA-DRB1*04:01*, in the conditional analysis of **bADA levels** in the **discovery + replication** pooled dataset of IFNβ-1b *s.c.*-treated patients.

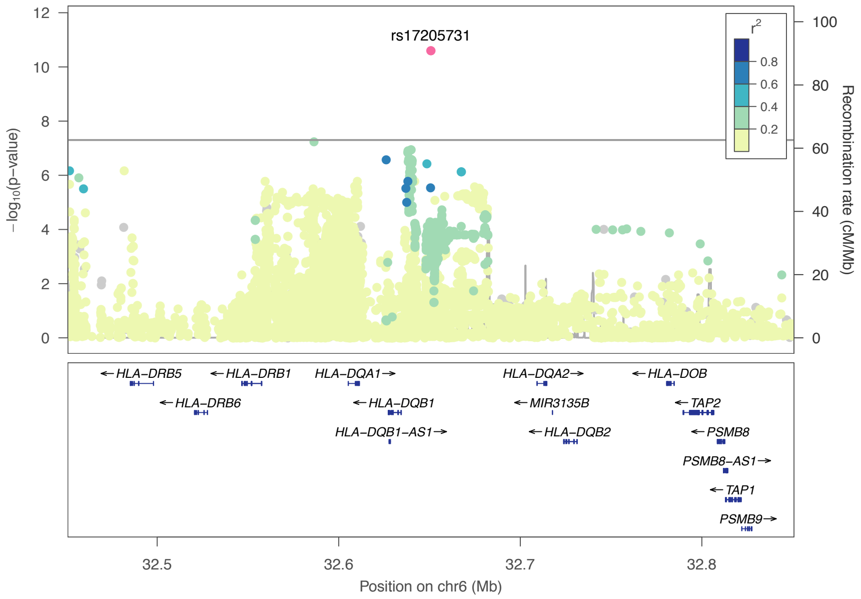

Supplement: Supplementary file 20 — Additional file 20. Regional association plots of the top GWAS variants in the analysis of IFNβ-1b s.c.-treated patients. Regional association plots of variants from the GWAS generated using LocusZoom v1.4 and the 1000 Genomes 1000G_Nov2014 EUR reference panel [72]. The color of dots indicates LD with the lead variant (pink). Gray dots represent signals with missing LD r2 values. If no LD information was present in the database on the top variant, LD with the variant showing the second-lowest p-value is indicated. The gray line indicates genome-wide significance. cM: centimorgan, chr: chromosome, Mb: mega base pairs. [file 12916_2020_1769_MOESM20_ESM.pdf]
