# Supplementary material for: Treatment- and population-specific genetic risk factors for anti-drug antibodies against interferon-beta: a GWAS
Source: BMC Med. 2020 Nov 4;18:298. doi: 10.1186/s12916-020-01769-6 (PMC7641861; doi:10.1186/s12916-020-01769-6)

Treatment-specific prediction of the presence of nADA in the replication data: performance of single models.

Eight PRS, the top single GWAS variant, and the top *HLA* allele from the discovery stage. Covariates: sex, age, treatment duration, titration site, and ancestry components. The plots show the area under the receiver operating characteristic curve (AUC) and its 95 % confidence interval (CI). Bonferroni = significant after Bonferroni correction for multiple testing; nominal = nominally significant (p<0.05); n.s. = not significant.

A: AUC for the prediction of the presence of nADA across all treatment preparations using all ten prediction models based on analyses for IFNβ (all preparations) in the discovery data.

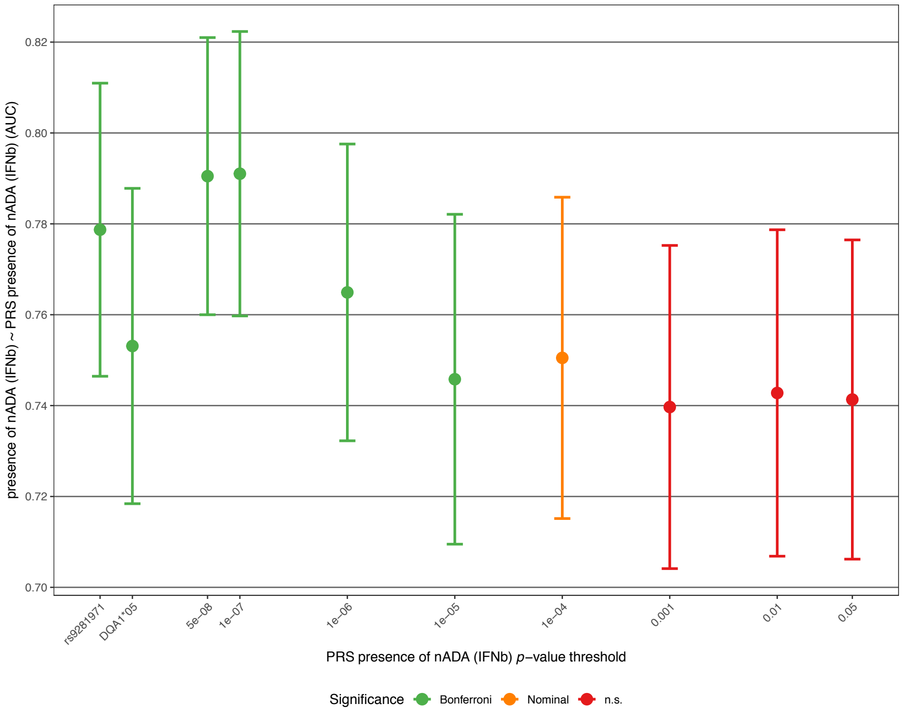

B: AUC for the prediction of the presence of nADA in IFNβ-1a *s.c.*-treated patients using all ten prediction models based on analyses for IFNβ (all preparations) in the discovery data.

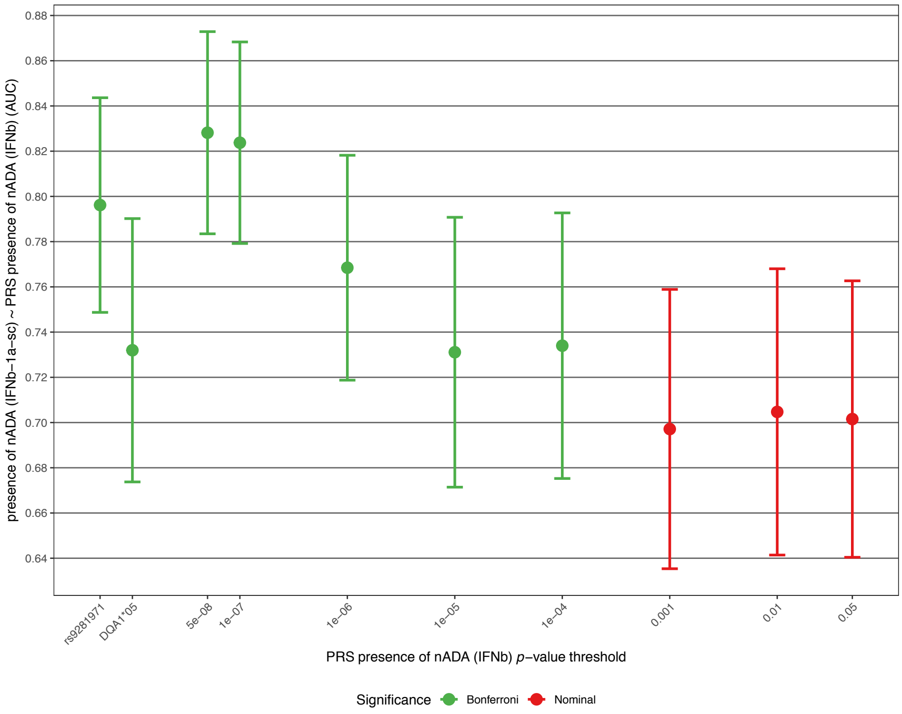

C: AUC for the prediction of the presence of nADA in IFNβ-1a *s.c.*-treated patients from the replication data using all ten prediction models based on analyses for IFNβ-1a (IFNβ-1a *s.c.* + IFNβ-1a *i.m.*) in the discovery data.

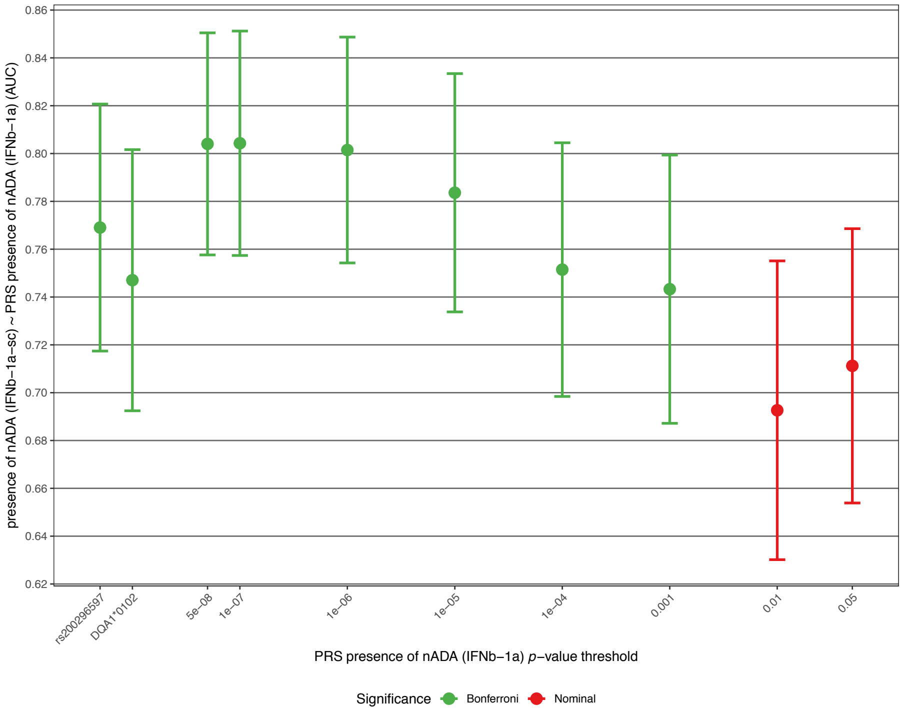

D: AUC for the prediction of the presence of nADA in IFNβ-1a *s.c.*-treated patients from the replication data using all ten prediction models based on analyses for IFNβ-1a *s.c.* in the discovery data.

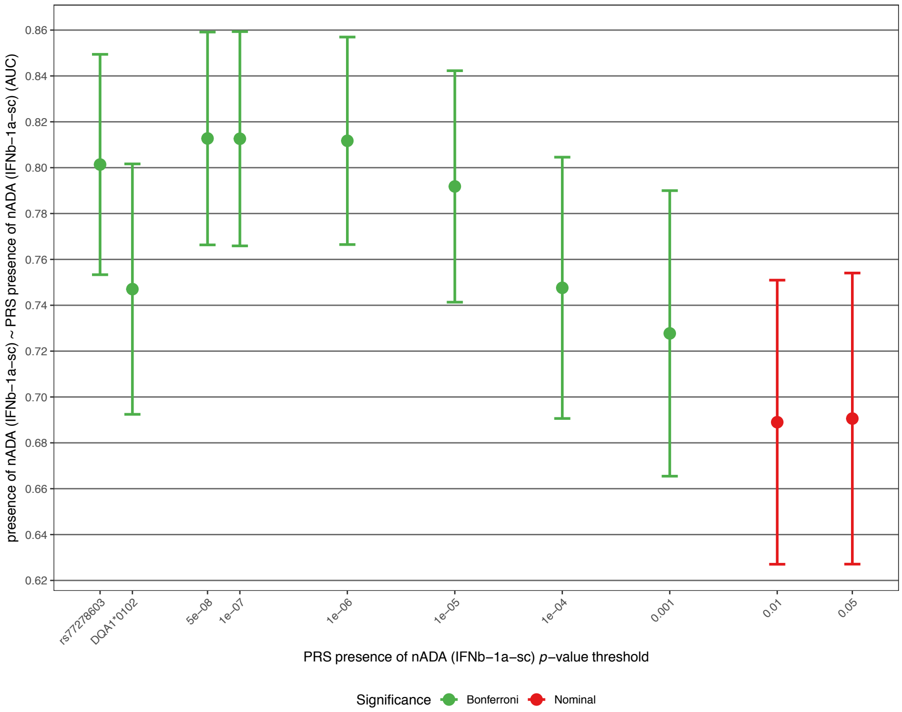

E: AUC for the prediction of the presence of nADA in IFNβ-1a *s.c.*-treated patients from the replication data using all ten prediction models based on analyses for IFNβ-1b *s.c.* in the discovery data.

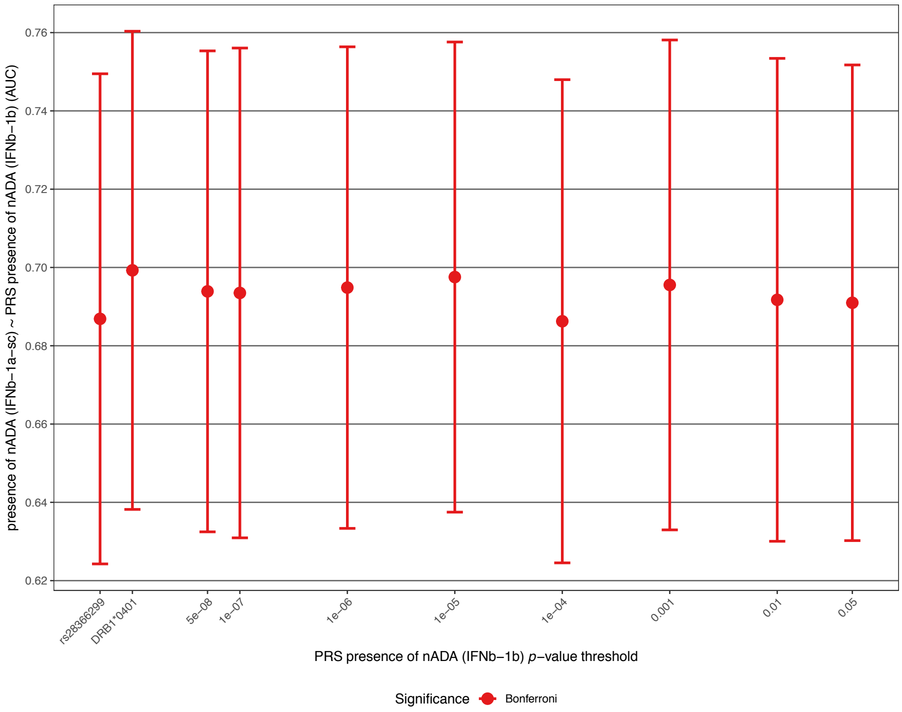

F: AUC for the prediction of the presence of nADA in IFNβ-1b *s.c.*-treated patients from the replication data using all ten prediction models based on analyses for IFNβ (all preparations) in the discovery data.

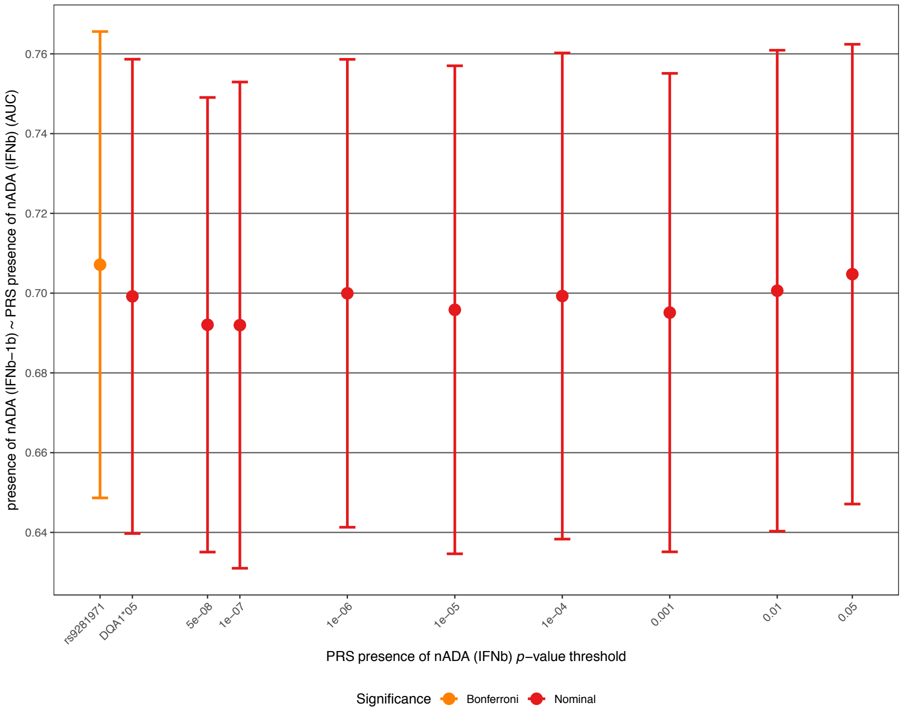

G: AUC for the prediction of the presence of nADA in IFNβ-1b *s.c.*-treated patients from the replication data using all ten prediction models based on analyses for IFNβ-1b *s.c.* in the discovery data.

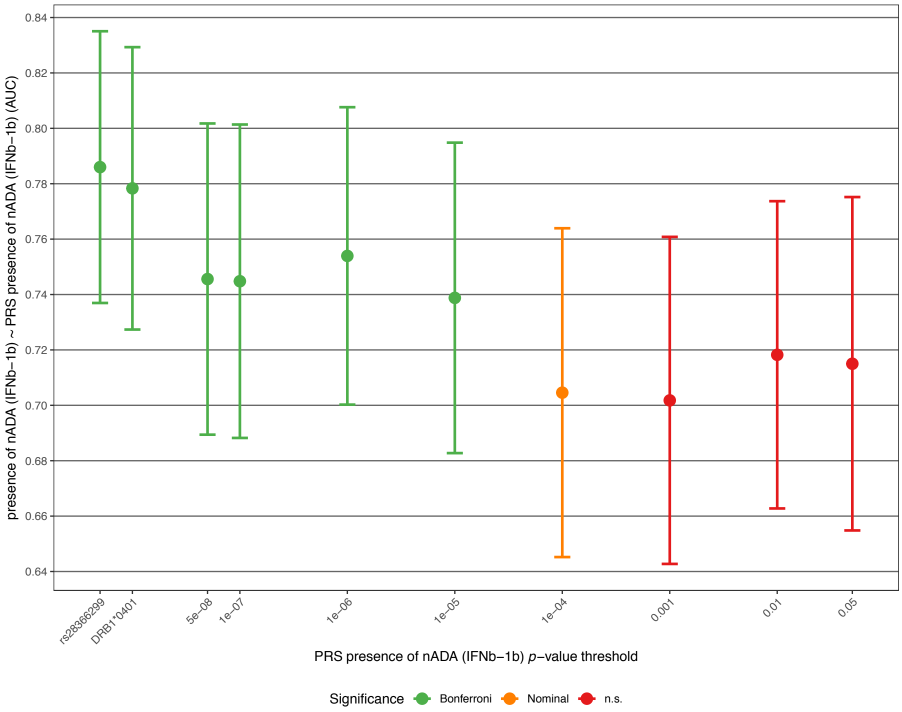

H: AUC for the prediction of the presence of nADA in IFNβ-1b *s.c.*-treated patients from the replication data using all ten prediction models based on analyses for IFNβ-1a *s.c.* in the discovery data.

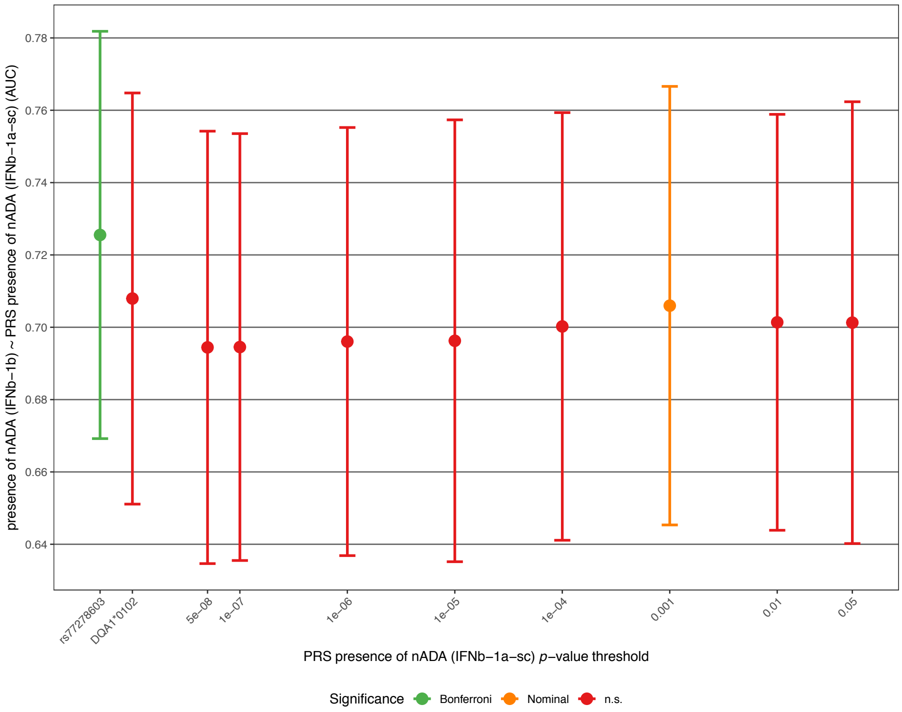

I: AUC for the prediction of the presence of nADA in IFNβ-1b *s.c.*-treated patients from the replication data using all ten prediction models based on analyses for IFNβ-1a (IFNβ-1a *s.c.* + IFNβ-1a *i.m.*) in the discovery data.

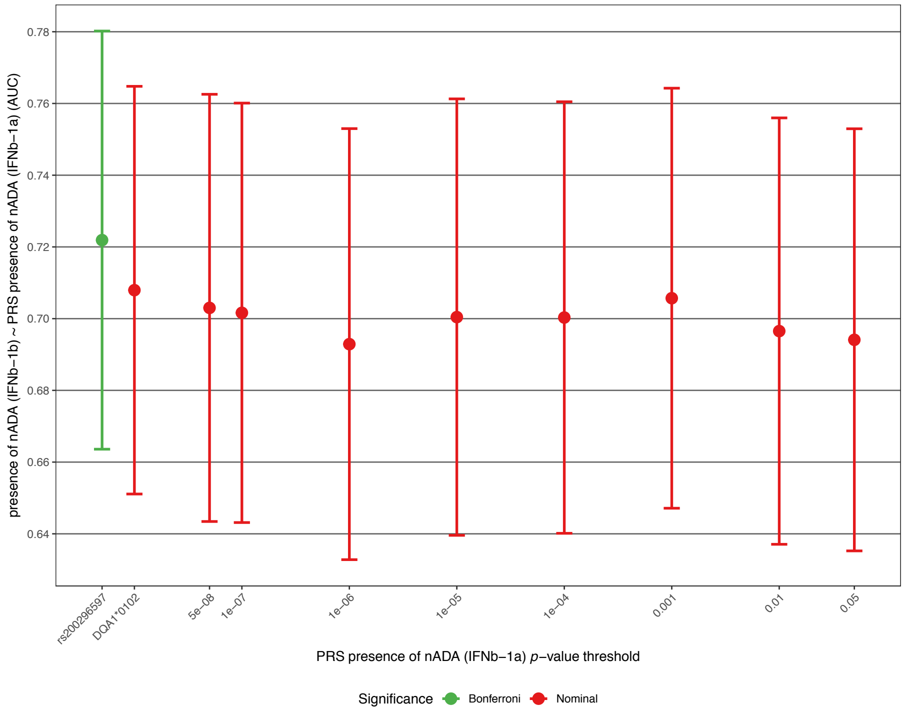

Supplement: Supplementary file 25 — Additional file 25. Treatment preparation-specific prediction of the presence of nADA in the replication data: performance of single models. Eight PRS, the top single GWAS variant, and the top HLA allele from the discovery stage. Covariates: sex, age, treatment preparation, treatment duration, titration site, and ancestry components. The plots show the area under the receiver operating characteristic curve (AUC) and its 95% confidence interval (CI). Bonferroni = significant after Bonferroni correction for multiple testing; nominal = nominally significant (p < 0.05); n.s. = not significant [file 12916_2020_1769_MOESM25_ESM.pdf]
