# Supplementary material for: Treatment- and population-specific genetic risk factors for anti-drug antibodies against interferon-beta: a GWAS
Source: BMC Med. 2020 Nov 4;18:298. doi: 10.1186/s12916-020-01769-6 (PMC7641861; doi:10.1186/s12916-020-01769-6)

Treatment-specific prediction of the presence of nADA in the replication data: comparison of top models.

For each top model, the plots show either the AUC and its 95 % CI or Nagelkerke's pseudo-R^2 and its 95 % CI. Boxes show the prediction groups (replication data) and columns within each box the training data groups (discovery data). Bonferroni = significant after Bonferroni correction for multiple testing; nominal = nominally significant (p<0.05); n.s. = not significant.

A: Prediction of the presence of nADA using analyses on the presence of nADA.
AUC of the model with the highest AUC for each treatment preparation.

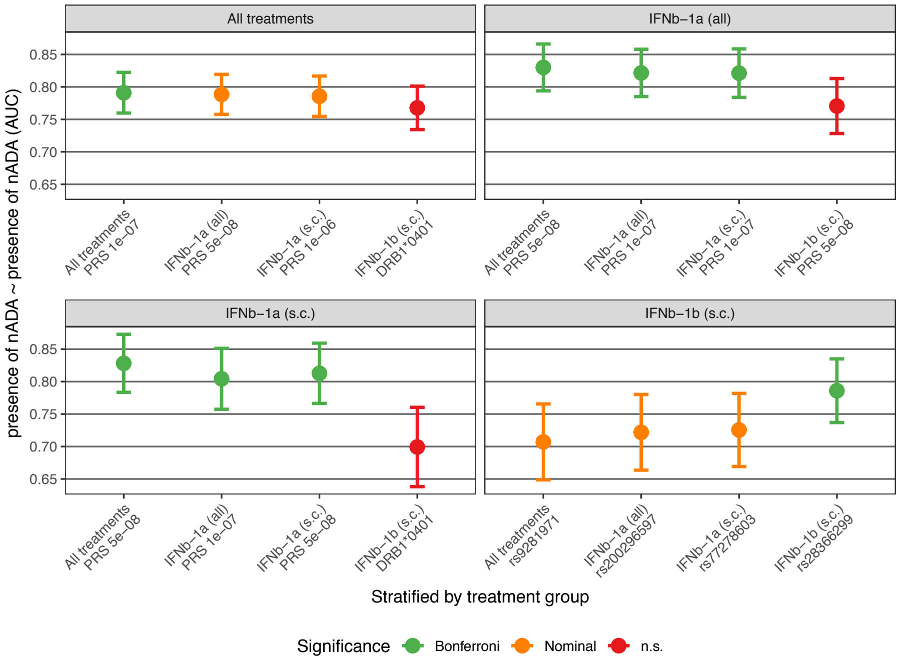

B: Prediction of the presence of nADA using analyses on the presence of nADA.
Nagelkerke's pseudo-R^2 of the model with the highest AUC for each treatment preparation.

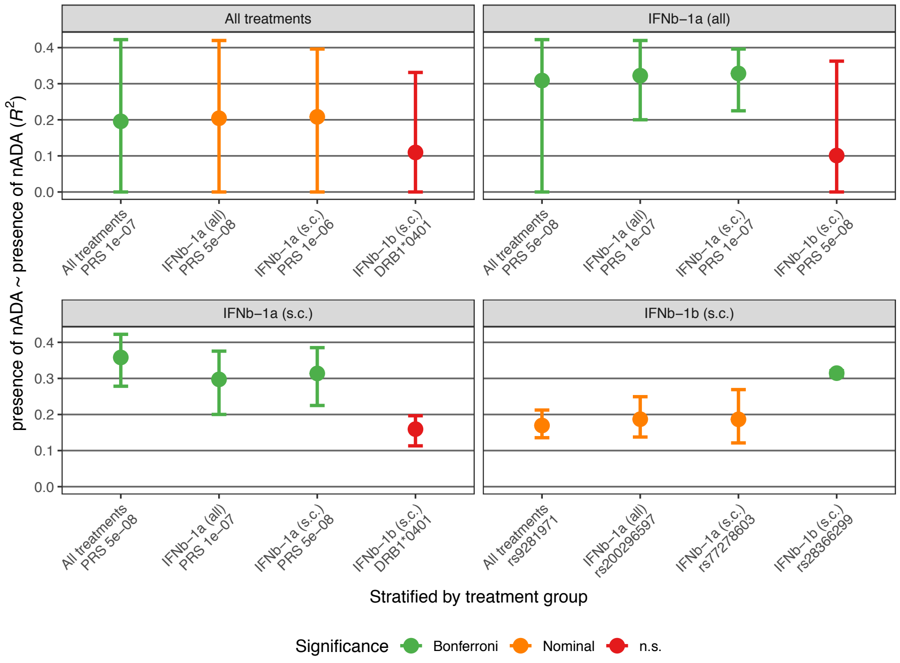

C: Prediction of the presence of nADA using analyses of nADA titers.
AUC of the model with the highest AUC for each treatment preparation.

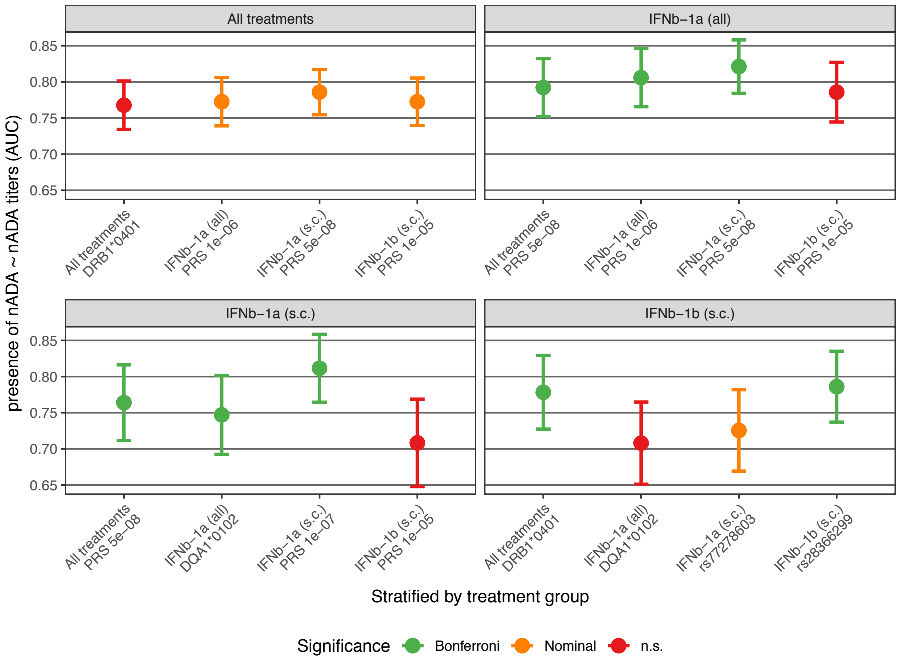

D: Prediction of the presence of nADA using analyses of nADA titers.
Nagelkerke's pseudo-R^2 of the model with the highest AUC for each treatment preparation.

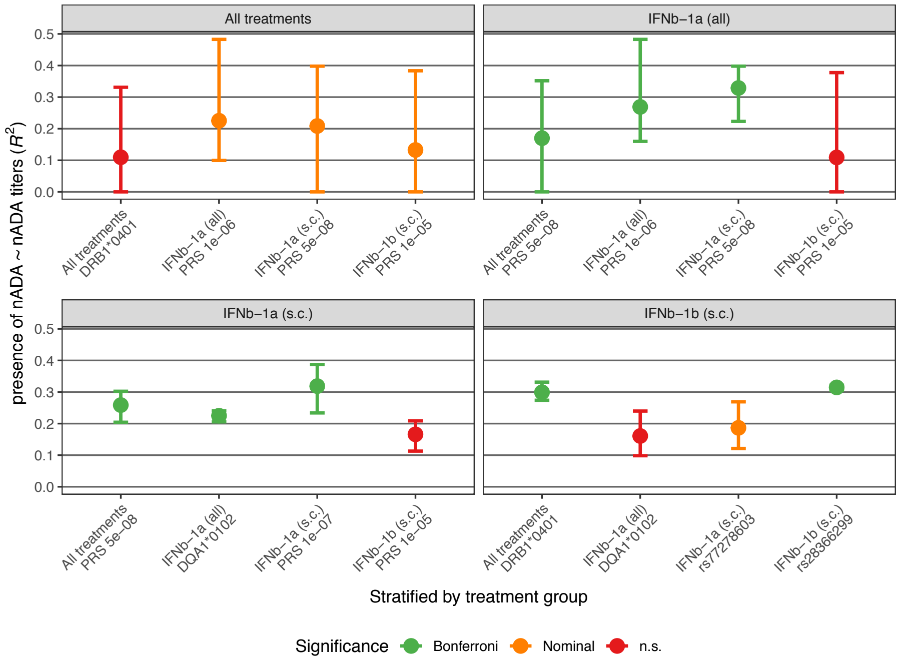

E: Prediction of the presence of nADA using analyses of bADA levels.
AUC of the model with the highest AUC for each treatment preparation.

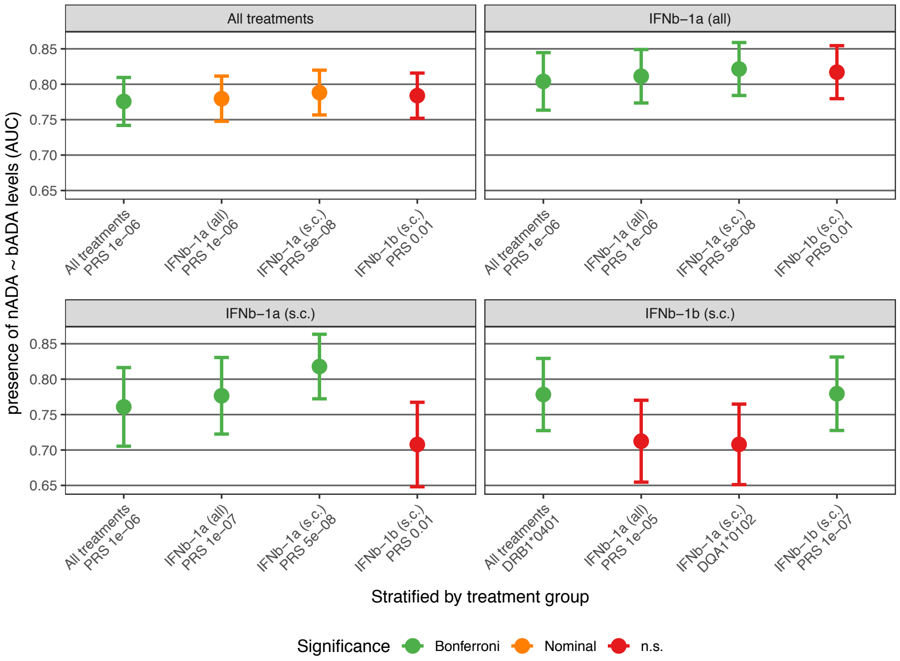

F: Prediction of the presence of nADA using analyses of bADA levels.
Nagelkerke's pseudo-R^2 of the model with the highest AUC for each treatment preparation.

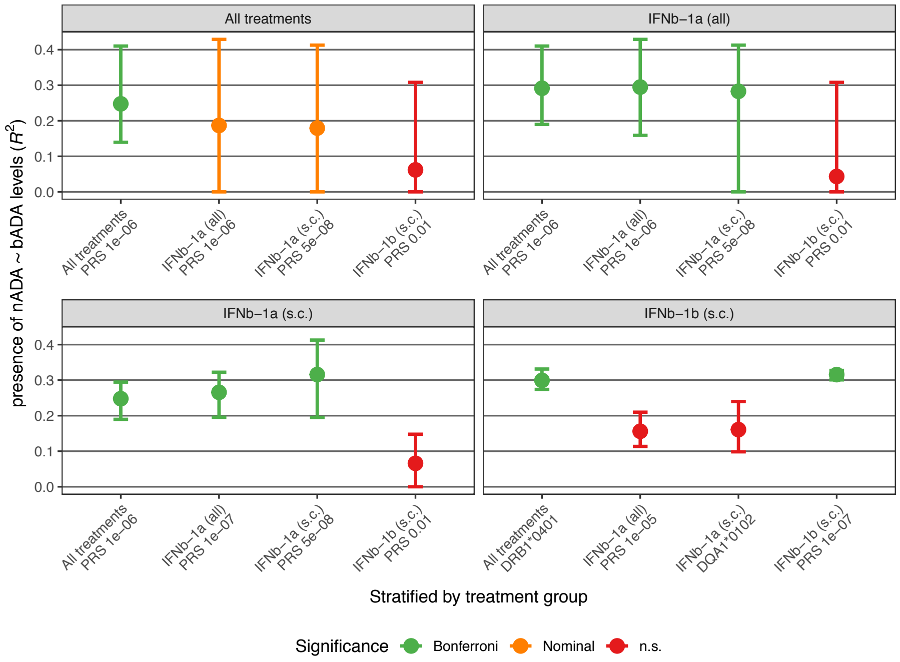

Supplement: Supplementary file 26 — Additional file 26. Treatment preparation-specific prediction of the presence of nADA in the replication data: comparison of top models. For each top model, the plots show either the AUC and its 95% CI or Nagelkerke’s pseudo-R2 and its 95% CI. Boxes show the prediction groups (replication data) and columns within each box the training data groups (discovery data). Bonferroni = significant after Bonferroni correction for multiple testing; nominal = nominally significant (p < 0.05); n.s. = not significant. [file 12916_2020_1769_MOESM26_ESM.pdf]
